# Integrating Environment and Aging Research: Opportunities for Synergy and Acceleration

**DOI:** 10.3389/fnagi.2022.824921

**Published:** 2022-02-21

**Authors:** Kristen M. C. Malecki, Julie K. Andersen, Andrew M. Geller, G. Jean Harry, Chandra L. Jackson, Katherine A. James, Gary W. Miller, Mary Ann Ottinger

**Affiliations:** ^1^Department of Population Health Sciences, School of Medicine and Public Health, University of Wisconsin-Madison, Madison, WI, United States; ^2^Buck Institute for Research on Aging, Novato, CA, United States; ^3^United States Environmental Protection Agency, Office of Research and Development, Durham, NC, United States; ^4^Division of National Toxicology Program, National Institute of Environmental Health Sciences, Durham, NC, United States; ^5^Division of Intramural Research, Department of Health and Human Services, Epidemiology Branch, National Institute of Environmental Health Sciences, National Institutes of Health, Durham, NC, United States; ^6^Department of Health and Human Services, National Institute on Minority Health and Health Disparities, National Institutes of Health, Bethesda, MD, United States; ^7^Department of Environmental and Occupational Health, Colorado School of Public Health, University of Colorado Denver, Denver, CO, United States; ^8^Department of Environmental Health Sciences, Columbia University Mailman School of Public Health, New York, NY, United States; ^9^Department of Biology and Biochemistry, University of Houston, Houston, TX, United States

**Keywords:** aging biology, gerotoxicology, toxicants, exposome, cumulative risk

## Abstract

Despite significant overlaps in mission, the fields of environmental health sciences and aging biology are just beginning to intersect. It is increasingly clear that genetics alone does not predict an individual’s neurological aging and sensitivity to disease. Accordingly, aging neuroscience is a growing area of mutual interest within environmental health sciences. The impetus for this review came from a workshop hosted by the National Academies of Sciences, Engineering, and Medicine in June of 2020, which focused on integrating the science of aging and environmental health research. It is critical to bridge disciplines with multidisciplinary collaborations across toxicology, comparative biology, epidemiology to understand the impacts of environmental toxicant exposures and age-related outcomes. This scoping review aims to highlight overlaps and gaps in existing knowledge and identify essential research initiatives. It begins with an overview of aging biology and biomarkers, followed by examples of synergy with environmental health sciences. New areas for synergistic research and policy development are also discussed. Technological advances including next-generation sequencing and other-omics tools now offer new opportunities, including exposomic research, to integrate aging biomarkers into environmental health assessments and bridge disciplinary gaps. This is necessary to advance a more complete mechanistic understanding of how life-time exposures to toxicants and other physical and social stressors alter biological aging. New cumulative risk frameworks in environmental health sciences acknowledge that exposures and other external stressors can accumulate across the life course and the advancement of new biomarkers of exposure and response grounded in aging biology can support increased understanding of population vulnerability. Identifying the role of environmental stressors, broadly defined, on aging biology and neuroscience can similarly advance opportunities for intervention and translational research. Several areas of growing research interest include expanding exposomics and use of multi-omics, the microbiome as a mediator of environmental stressors, toxicant mixtures and neurobiology, and the role of structural and historical marginalization and racism in shaping persistent disparities in population aging and outcomes. Integrated foundational and translational aging biology research in environmental health sciences is needed to improve policy, reduce disparities, and enhance the quality of life for older individuals.

## Introduction

By 2050, one in five adults living in the United States will be over the age of 65 years. It is, therefore, of concern to determine how exposure to environmental factors encountered over the life span might influence the aging trajectory and affect the age-related health of individuals. How the aging process influences response to environmental toxicants and other external stressors is also of concern. Health scientists have studied the impact of environmental toxicants on age-related diseases such as cardiovascular disease, metabolic disorders, cancer, and neurological disease as well as the role of genetic predisposition (Bektas et al., 2018; Franceschi et al., 2018). This research has identified an intersection between toxicant exposure and age-related health outcomes. Environmental health research has also identified several socioeconomic and societal factors that intersect with these adverse exposures and contribute to aging-related health inequalities among individuals and between communities.

Biological aging is closely related to environmental toxicity, starting with an individual’s diminished ability to metabolize, compensate, and recover from exposure to adverse stressors, all of which can lead to altered homeostasis of key biological systems (Bektas et al., 2018; Franceschi et al., 2018). Thus, aging processes and chronological age must be considered in assessing both how and when exposures become toxic and if there are cumulative effects that make the individual more vulnerable. Moreover, we know that other external environmental stressors experienced during the lifetime vary across individuals and populations. These stressors are diverse and include unstable, insecure, or unsafe housing conditions, food insecurity, or adverse experiences including trauma and discrimination. We are now beginning to appreciate how such stressors combined with environmental toxicants can impact human health, the aging process, and reflect age-related social vulnerability. For example, the SARS-COV-2 pandemic uncovered how environmental factors, biological phenotypes, and situational stressors can shape a population’s biological susceptibility and social vulnerability leading to an unequal burden of morbidity and mortality (Petroni et al., 2020; Wu et al., 2020; Bourdrel et al., 2021).

Recent advances in our understanding of the normal aging process, the biological susceptibility of the aged population, and assessments within environmental health sciences set a solid framework to support and advance inter- and *trans*-disciplinary research efforts. Such collaborations are in their infancy but offer great potential. We acknowledge that aging is a complex physiological phenomenon that occurs across the lifecourse. Among the many factors contributing is antagonistic pleiotropy an evolutionary mechanism of aging—with a genetic trade-off over the lifespan that may also influence how environmental toxicant exposures alter the aging process leading to variable exposure response relationships between individuals and across populations (Austad and Hoffman, 2018; Mitteldorf, 2019; Byars and Voskarides, 2020). Research using systems biology approaches indicate that environmental and aging exposures appear to affect certain common key biological processes. These commonalities should facilitate a cross-integration of the two disciplines. Additionally, such research can provide an opportunity to inform novel programs and policies to improve population health and address disparities.

**FIGURE 1:**
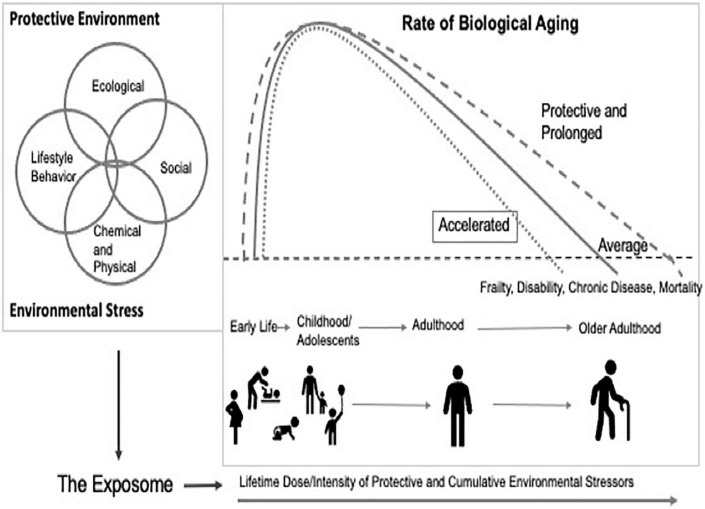
Integrated aging and environmental health research.

In June of 2020, the National Academies of Science and Engineering Standing Committee on the Use of Emerging Science for Environmental Health Decision-Making convened a workshop that focused on bridging gaps in aging research and environmental health sciences. The workshop entitled, “Integrating the Science of Aging and Environmental Health Research” convened multi-disciplinary researchers with expertise across exposure science, epidemiology, sociology, molecular biology, genetics, toxicology. and biological aging to identify knowledge gaps and research needs. The deliberations highlighted numerous opportunities to advance mechanistic understanding of the role of the environment in health and aging, while appreciating how the trajectory of aging is shaped by the broader intersection of environmental exposures. Figure 1 represents a framework that recognizes lifetime exposure to toxicants as contextually driven, acute or cumulative, dynamic, and often highly correlated with other external social stressors.

In the sections that follow, we broadly review key biological mechanisms in environmental health and aging research that could support and accelerate focused multi- and transdisciplinary partnerships. In addition to the review of key biological mechanisms of aging, we discuss advances in our understanding of the independence and intersection of environmental influences on aging processes, using examples of traditional chemical exposures as well as a more comprehensive framework that considers the totality of environmental exposures (chemical and non-chemical) (Agache et al., 2019; Niedzwiecki et al., 2019; Walker et al., 2019; Cheung et al., 2020). We end with a vision of how integration of environmental health sciences with aging biology research can help guide effective research and policy translation.

## The Biology of Aging

**FIGURE 2:**
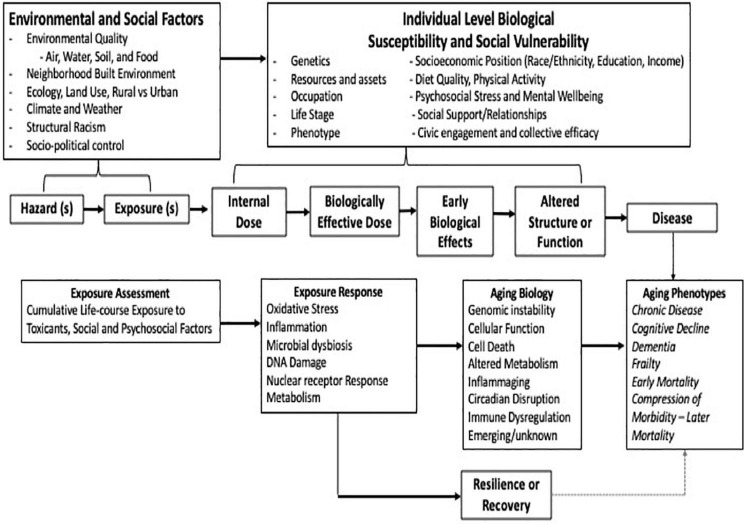
Integrating the science of aging and environmental health.

Aging is a sequence initiated during development that sets the framework for later events (Barker et al., 1989; Wright, 2017; Ferrucci et al., 2020). The accumulation of different types of factors—biological and environmental—across the life span can modify this framework and shift or alter ongoing adaptations to age-related challenges. This can result in increased age-related biological susceptibility and/or damage later in life. The “Geroscience Hypothesis” states that strategies designed to modify biological drivers of aging will slow the progression of aging and prevent or delay the onset of chronic disease (Figure 2; Kohanski et al., 2016; Sierra, 2016a,b; Guerville et al., 2020). Within that same concept falls the idea that modifying factors, such as genetic, physiological, social, and environmental, can accelerate the process and/or diminish health status. A major premise of the hypothesis is that biological resilience represents an organism’s ability to successfully respond and adapt to challenging life conditions to maintain homeostatic balance in the face of damaging stress. This resilience is challenged by aging (Geronimus et al., 2006; Geronimus et al., 2010; Ferraro et al., 2017). Associated biological processes are highly effective at maintenance and repair early in life (Kirkwood, 2005). This is followed by a period of relative stability, becoming less so with age-related reductions in reserve biological capacity. An inflection point is reached when the ability of the body to compensate is no longer sufficient to maintain organismal homeostasis and health. As unrepaired damage accumulates beyond a functional threshold for repair, adverse health effects become evident (Ferrucci and Fabbri, 2018; Franceschi et al., 2018; Ferrucci et al., 2020).

The complex aging process represents a set of fundamental and interconnected biological processes, sometimes referred to as “hallmarks of aging” (Lopez-Otin et al., 2013; Guerville et al., 2020). The culmination of structural and functional changes that occur require continual adaptation that results in the gradual accumulation of molecular and cellular deficiencies or damage. The accumulating damage shifts normal immune and metabolic homeostasis, increasing potential for numerous aging-related diseases, frailty, and early mortality (Bektas et al., 2018; Franceschi et al., 2018). Critical biological processes associated with aging include genomic instability, telomere attrition, epigenetic alterations, loss of proteostasis, altered metabolism, mitochondrial dysfunction, cellular senescence, stem cell exhaustion, altered intercellular communication, macromolecular damage, and chronic low-grade inflammation.

### Genomic Instability

Genomic instability, represented as DNA somatic mutation accumulation and the loss of efficient DNA repair mechanisms, has been proposed as an important driver of biological aging. DNA strand breaks resulting in epigenetic changes, transcriptional alterations, and telomere attrition are indicative of changing DNA repair mechanisms (Saha et al., 2008; Collins, 2014). Examination of telomere length as a molecular clock is supported by the observation of shortening of telomere length with age and thus, diminished protection from DNA damage response. Telomere length tends to be extremely heterogeneous across individuals, thus a challenge in biomarker development (Lin et al., 2015; Puterman et al., 2015). Interestingly, changes observed in newborn telomere length may reflect the fetal and maternal environment (Mainous et al., 2014; Bosquet Enlow et al., 2019; Colicino et al., 2020; Cowell et al., 2020; Lee et al., 2020).

### Epigenetics

Epigenetics refers to the ensemble of mechanisms that define stable phenotypic characteristics and can modulate gene expression programs in response to environmental cues. Epigenetic modifications include DNA methylation, histone modification, chromatin remodeling, and non-coding RNA (Pal and Tyler, 2016). DNA methylation is easily assessed in circulating cells and is relatively stable over time, allowing for examination across aging and in age-related chronic diseases (Gensous et al., 2017; Belsky et al., 2018; Levine et al., 2018). During early-life periods, epigenetic mechanisms refine the genetic program that is responsive to environmental challenges; which is followed by continuous epigenetic tuning over the life span (Brieno-Enriquez et al., 2015). Epigenetic tuning is involved in multiple aspects of aging biology, including genetic repair mechanisms, inflammation and immune homeostasis, and receptor mediated responses. Changes may be adaptive or conversely contribute to adverse responses in later life, resulting in chronic disease or early mortality (Barker et al., 1989; Wadhwa et al., 2009; Pembrey et al., 2014; Ben-Shlomo et al., 2016). Several “epigenetic clocks” derived from a constellation of DNA methylation sites that track with multiple indicators of aging related biomarkers of disease and with chronological aging have been used to assess differences between chronological and biological age (Hannum et al., 2013; Horvath, 2013; Knight et al., 2016; Maierhofer et al., 2017; Sehl et al., 2017; Levine et al., 2018; Lu et al., 2019). These clocks have become increasingly used to estimate resilience in responding to the long-term influence of environmental exposures (Nwanaji-Enwerem et al., 2016; Dhingra et al., 2018; Gao et al., 2018; Nwanaji-Enwerem et al., 2020; Nwanaji-Enwerem et al., 2021b).

### Proteostasis, Mitochondrial Dysfunction, and Cellular Senescence

Alterations in cellular processes such as proteostasis and cellular senescence also occur during aging. Proteostasis is a process by which the organism repairs, recycles, and eliminates damaged macromolecules to maintain cell integrity and function (Cuervo et al., 2005; Cuervo and Macian, 2014; Zhang et al., 2016). This becomes greatly reduced during the aging process. The accumulation of damaged mitochondria and mitochondrial DNA during aging can also reduce energy availability and increases levels of reactive oxygen species leading to macromolecule damage (Elfawy and Das, 2019). Changes in mtDNA copy number and degree of heteroplasmy in human blood cells and tissue biopsies provide information on mitochondrial physiology relevant to aging and age-related diseases (Zhang et al., 2017; McDermott et al., 2018; Moore et al., 2018; Vaz Fragoso et al., 2019). Cellular senescence is a stress response mechanism involving replication arrest and other complex cellular changes (Rodier and Campisi, 2011; Munoz-Espin and Serrano, 2014; Song et al., 2020). The accumulation of senescent cells and negative effects of associated proteins on cell matrix and progenitor cells can contribute to tissue degeneration and dysfunction. Related blood biomarkers are based on the assumption that the associated proteins in tissue are released into the circulation (Bernardes de Jesus and Blasco, 2012).

### Inflammation

Inflammation is a common hallmark of numerous age-related diseases (Hood and Amir, 2017b; Furman et al., 2019) manifesting as chronic low-grade systemic inflammation or “inflammaging” (Franceschi and Campisi, 2014; Fulop et al., 2017; Ferrucci and Fabbri, 2018). Inflammaging also encompass alterations in mitochondrial function and dysregulation of the inflammatory response (Hood and Amir, 2017b; Costantini et al., 2018; Furman et al., 2019). As one example, the inflammasome pathway represents a complex process that is sensitive to stress/danger signals and may shift in responsivitiy over the life-time (Swanson et al., 2019). Several nuclear receptors involved in environmental metabolism, including the aryl-hydrocarbon receptors (AhR), have been cited as important regulators of immune homeostasis leading to chronic inflammation; these responses are often orchestrated at the cellular level (Liu et al., 2017; Guarnieri et al., 2020). Thus, a functional compromise in immune cells and/or altered immune homeostasis, either by dysregulation or immunosenescence, can lead to compromised elimination of pathogens, pre-malignant cells, senescent cells, cellular debris, and/or aberrant proteins from the tissue leading to their toxic accumulation.

### Gut Microbiome

Microorganisms (microbiota) colonize the intestinal tract, generally believed to start after delivery, with a shift in composition and abundance of individual microbiota occurring to varying degrees through adulthood, presumably reflecting differences in lifestyle, environmental, race/ethnicity, and genetics (Lozupone et al., 2012; Gilbert et al., 2018). The gut microbiome plays a key role in regulating and maintaining host metabolism and immunity, with emerging evidence suggesting connections between significant shifts in gut dysbiosis (a major shift in composition) and development of numerous age-related diseases (Shiels et al., 2019; Castro-Mejía et al., 2020; Ragonnaud and Biragyn, 2021; Wilmanski et al., 2021). Gut dysbiosis has been linked to decline, and other neurological outcomes (Gareau, 2016; Nagpal et al., 2018; Wilmanski et al., 2021). The gut microbiome is impacted by nutritional factors and diet and age or disease-associated changes can alter general metabolic processes as well as influence the level of environmental chemicals circulating in the body and how they are excreted. Current data suggests that environmental factors can contribute to alterations in gut microbiome dysbiosis and indicate that gut microbiome composition can influence toxicant or pharmaceutical metabolism (Wilson and Nicholson, 2017), as well as be modified by such exposures (He et al., 2020; Tu et al., 2020). With age, alterations in the gut microbiome are associated with inflammation, immunomodulation, modified metabolism, changes in receptor-mediated responses, epigenetic alterations, and general gut leakiness (Kim and Jazwinski, 2018; Sharma et al., 2020; Conway, 2021). We now know that the state of the gut microbiome can be modified over time through external factors and significantly influences multiple organ systems including the nervous system (Castro-Mejía et al., 2020; Cryan et al., 2020). However, the mediating role of the microbiome in the association between environmental pollutants and aging remains unclear. Most evidence for environmental toxic effects on the microbiome comes from animal studies (Tu et al., 2020). The limited number of human studies leaves many questions unanswered.

### Circadian Rhythms

Circadian rhythm homeostasis serves to support healthy aging and numerous aspects of aging biology (Judge et al., 2017). Circadian rhythms adapt and coordinate critical physiological functions and cellular processes within a defined time cycle. The age-associated circadian profile reflects not only timing but temperature rhythms, circulating levels of melatonin, cortisol and steroid hormones, inflammatory processes, and the expression of clock genes (Lamont et al., 2007; Roenneberg et al., 2007; Sitzmann et al., 2010; Urbanski and Sorwell, 2012; Scheiermann et al., 2013). Age-associated disruptions in circadian rhythms include altered sleep manifested as shifts in sleep cycles, tolerance for cycle change, or disrupted sleep timing (Bliwise et al., 2005; Schmidt et al., 2012; Hood and Amir, 2017a), as well as altered diet and physical activity (Wehrens et al., 2017; Queiroz et al., 2020). These behavioral disruptions can affect a range of physiological responses that can result in functional alterations at the molecular and cellular levels (Schmidt et al., 2012; Hood and Amir, 2017b).

### Intersecting and Overlapping Mechanisms Provide Valuable Insights and Approaches

Aging research aims to better understand multiple molecular and biological pathways affecting the rate of aging in populations, but a more comprehensive systems biology approach is needed. Of the numerous biomarkers of aging, mitochondrial function, DNA methylation, telomere length, gut microbial dysbiosis and possibly cellular senescence and autophagy are at a stage to be implemented in larger epidemiological studies, with the caveat of attention to limitations and details for assaying each. However, none of the measures described represent a full picture of biological aging but rather one component of a panel of various biomarkers that reflect cumulative damage across regulatory systems. Approaches that leverage across various molecular pathways and biomarkers show the greatest promise for future evaluations of the impact of environmental exposures on the aging process and on age-related susceptibility (Geller and Zenick, 2005; Gensous et al., 2017; Ahadi et al., 2020).

Many of our insights into the fundamentals of aging processes have been gained from studies on the comparative biology of aging. Some notable species, such as the naked mole rat, show negligible senescence during aging, often accompanied by specific adaptations such as resistance to oxidative stress (Ma and Gladyshev, 2017; Saldmann et al., 2019). These adaptations are also observed in non-mammalian species, with comparison of short- and long-lived species such as in birds (Ogburn et al., 2001). Furthermore, short-lived species show many age-related declines in physiological systems that are seen in mammals, as well as elevated disease incidence, including cancer (Gorham and Ottinger, 1986; Ottinger et al., 2004; Ottinger and Lavoie, 2007; Johnson and Giles, 2013). As such, these comparative studies offer significant insights into conserved mechanisms occurring throughout vertebrates during the aging process (National Research Council, 2014; Niedernhofer et al., 2017).

### Aging, Phenotypes, and Outcomes

The consequence of shifts in aging biology over time is ultimately a set of multi-comorbid age-related diseases (Fried et al., 2001; Elliott et al., 2021), including cancer, cardiovascular disease, metabolic disorders (e.g., diabetes), and neurodegenerative disorders. A culmination of chronic conditions and biological shifts occurring with aging contribute to what is often referred to as “frailty.” From a biological perspective, frailty may be defined an outcome of several systemic changes, including altered metabolism, homeostatic regulation, low-level inflammation, and neurological deficits (Walston et al., 2002; Franceschi and Campisi, 2014; Bektas et al., 2018). These systemic changes contribute to a phenotype of decreased physiological ability manifested as gait speed and overall low physical activity (Bortz, 2002; Apóstolo et al., 2017), which are associated with higher risk for falls, disability, hospitalizations, and mortality (Fried et al., 2001; Searle et al., 2008; Sternberg et al., 2011). Frailty is also associated with reduced pharmacokinetic and pharmacodynamic functions (Walston et al., 2002; Crome, 2003; Kinirons and O’Mahony, 2004). Frailty indices often used in clinical and community settings assess physical, psychological, and social functioning by examining activities of daily living, cognition, mood, and physical performance (Johnson et al., 2014; Apóstolo et al., 2017). Hearing and vision deficits may also be considered with other age-related co-morbidities (Apóstolo et al., 2017).

## Aging Biology and Environmental Toxicants

Much work on the impacts of environmental toxicants on aging biology has focused on understanding the exacerbation of chronic cardio-metabolic, respiratory, and cancer risk with recent evidence showing impact on neurological and cognitive functions. Many of the current research efforts integrate an assessment of key disease-related mechanisms and pre-clinical indicators. Figure 2 presents the most prominent toxicological paradigm used in environmental sciences and the intersection with interim biomarkers of biological aging, a broader consideration of resilience and recovery, followed by potential decline. More recent thinking has developed a much broader perspective on what “environment” encompasses reflecting a composite of multiple, interrelated external factors, including chemical and physical environments and modifying social factors. A similar evolution in aging research has occurred. With the advancement of our understanding of key underlying biological mechanisms of aging, one can now consider the integration of new metrics into population health research (Lopez-Otin et al., 2013; Ferrucci and Fabbri, 2018; Franceschi et al., 2018; Guerville et al., 2020).

Our understanding environmental exposures aging biology have been augmented by studies on the comparative biology of aging. Some notable species, such as the naked mole rat show negligible senescence during aging, often accompanied by specific adaptations such as resistance to oxidative stress (Ma and Gladyshev, 2017; Saldmann et al., 2019). These adaptations are also observed in non-mammalian species, with comparison of short- and long-lived species such as in birds (Ogburn et al., 2001). Furthermore, short-lived species show many age-related declines in neural and endocrine systems and increased incidence of cancer (Gorham and Ottinger, 1986; Ottinger et al., 2004; Ottinger and Lavoie, 2007; Ottinger, 2007; Johnson and Giles, 2013). As such, these comparative studies offer significant insights into conserved mechanisms occurring throughout vertebrates during the aging process (National Academy of Sciences, 2014; Niedernhofer et al., 2017).

Differential individual resilience may also relate to lifetime exposures to contaminants and other environmental stressors. This is an essential component of the Exposome concept in which there is consideration of cumulative impacts of exposure to contaminants and other environmental stressors (Wild, 2012; Wild et al., 2013; Juarez et al., 2014; Cifuentes et al., 2019; Juarez et al., 2020a). Often it appears that early life exposures may have more severe effects both for the immature organism and over that individual’s lifespan. A case in point is the impact of exposure to polychlorinated biphenyls (PCBs) in the avian embryo, which result in non-lethal cardiac defects in hatchlings (Carro et al., 2013). Recent meta-analyses among human cohorts show similar cardiac defects at birth among humans are associated with *in utero* exposure to a range of cumulative environmental chemical exposures including pesticides, solvents, metals, and air pollutants (Nicoll, 2018). These studies reveal conserved mechanisms across species that provide a potential suite of bioindicators for effects of environmental stressors on aging processes.

Evidence from toxicology, comparative biology, and epidemiology demonstrate an association between environmental toxicant exposures and numerous age-related outcomes. Much of what we know about biological susceptibility to environmental toxicant exposure comes from children’s environmental health and is now being applied to other susceptible populations like the aged population (Craven et al., 2021; Shiels et al., 2021). Exposure and response relationships can vary based on acute, short term or long-term exposures and are dependent on the chemical structure and physiological properties of toxicants. Toxicant exposures early in development (*in utero*, or throughout childhood) are often greater in dose per body weight than in the adult, and can set in motion a cascade of altered gene expression, cellular, and other physiological processes that result in adverse effects that are not observed until later in life (Brieno-Enriquez et al., 2015; Martos et al., 2015; Nawrot et al., 2018; Ladd-Acosta et al., 2019; Smith et al., 2020). The release of tissue-sequestered toxicants at different stages of life can expose an individual to a mixture of compounds stored in the tissue during sensitive developmental stages. Alternatively, changes in metabolism or processing of essential metals and nutrients that occur with age can impact the uptake and metabolic processing of environmental chemicals.

Environmental exposures occur through several different routes including inhalation, dermal and ingestion. Each source and route of exposure may play a different role in aging biology. Dietary factors, for example, play an important role in both aging and environmental sciences. Diet quality is not only an important modifier of physiology, including gut microbiome composition which may affect detoxification. Diet is also often a source of toxicant exposure (Roy et al., 2003; Wang et al., 2019) and has been shown to alter circadian rhythm homeostasis (Gundert-Remy et al., 2015; Craven et al., 2021; Shiels et al., 2021). Diet is often linked with other lifestyle, behavioral or social factors that contribute to increased vulnerability to multiple sources of toxicants and social stressors simultaneously. This is particularly true for individuals and populations who have fewer resources and are in lower socio-economic strata. Socioeconomic position, limits access to resources and assets such as high quality food, and this is compounded by external neighborhood stress from both chemical and non-chemical sources, all of which combined can alter aging biology.

Numerous examples from epidemiologic or toxicological research where significant overlap between toxicity endpoints and key markers of biological aging exist. Opportunities for the future lie with the integration across the fields (Table 1; Peters et al., 2021). The more established examples for environmental exposures linked to effects on the aging process include air pollution, heavy metals, and pesticides. All three are associated with numerous aging related outcomes, including cardiometabolic disease (Moon et al., 2012; James et al., 2013; Tellez-Plaza et al., 2013; James et al., 2015; Lentini et al., 2017), cancer (Hayes, 1997; Nawrot et al., 2006; National Research Council, 2013), osteoporosis (James and Meliker, 2013), and neurological disorders (Power et al., 2014). Inflammaging and genomic instability are examples used to illustrate the overlapping mechanisms between environmental health and aging research, and to highlight opportunities for advancing interdisciplinary environmental health and aging research.

**TABLE 1:** Examples of the intersection between environmental toxicology mechanisms of action, aging biomarkers and aging related phenotypes.

Biology of aging	Environmental pollutants/classes (examples)	Aging biomarkers	Aging phenotypes*
*Genomic instability* Epigenetics Telomere attrition, STEM cell exhaustion	Air pollution Metals Pesticides Endocrine disruptors (plastics etc.) Polycyclic aromatic hydrocarbons	DNA methylation, chromatin and histone modification Non-coding and microRNA DNA strand breaks Epigenetic clocks Autophagy Receptor signalins mRNA changes	Cancer Cardiovascular health Chronic obstructive pulmonary disease Metabolic health Endocrine dysfunction Immune dysregulation
	
*Altered cellular processes* Proteostasis Cellular senescence/signaling Mitochondrial function	Air pollutants Metals PAHs	MtDNA copy number Reactive oxygen species Senescent cells, progenitor cells, Proteomic profiles Altered cellular matrix	Neurocognitive decline Parkinson’s Mild cognitive impairment Alzheimer’s disease
	
*Altered metabolism* Nutrient sensing	Heavy metals Endocrine disruptors (plastics etc.) Air pollution	Gut microbiome dysbiosis Metabolite changes Lipodomic profiles Proteomic profiles	Frailty Early mortality Compression of morbidity Later mortality
	
*Inflammation*	Air pollution Metals Pesticides Endocrine disruptors (plastics etc.) Polycyclic aromatic hydrocarbons	Inflammatory cytokeines Toll like receptors Nuclear receptor signaling/gene expreession Reactive oxygen species	*Select
	
*Circadian disruption*	Sleep/wake/light Diet/physical activity Occupation	Altered gene expreesion Metabolic and immune dysregulation	
			

*All phenotypes have been associated with aging biology and environmental stressors.

### Inflammaging

Environmental toxicant exposures often induce systemic inflammation and oxidative stress, which impact numerous aging processes. Many diseases are associated with changes in DNA, cellular proliferations, epigenetic regulation, microbiome function, shifts in metabolism and altered immune system homeostasis. In many cases, common age-related changes become accelerated and increasingly dysregulated. Recent research has implicated an environmental contribution to the process of inflammaging across multiple organs including the nervous system (Pezzoli and Cereda, 2013; Tellez-Plaza et al., 2013; Lucchini et al., 2019; Finch and Morgan, 2020) Chronic low-grade sterile inflammation, when left unchecked may manifest as immune-system impairment and loss of resilency.

A well-established body of literature supports the contribution of short-term and chronic air pollution exposures to age-related diseases, including cardio-metabolic and respiratory diseases, premature mortality, and increased risk for viral and other infectious diseases including Sars-COV-2 (Brunekreef and Holgate, 2002; Brugha and Grigg, 2014; Doiron et al., 2019; Wu et al., 2020; Khomenko et al., 2021). Repeated exposures over long periods of time leads to chronic systemic inflammation which results in accelerated cellular proliferation, protein and DNA damage, and diminished repair mechanisms (Brugha and Grigg, 2014; Ward-Caviness et al., 2016; Vriens et al., 2019). Air pollution contains a mixture of environmental toxins—including particulate matter, black carbon, ozone, and secondary nitrates, which vary by sources (e.g., agriculture, mining or other industry, traffic). Assessment of such mixtures has shown that particulate matter (PM_2.5_) can penetrate deep into lungs and activate a complex immune response, which induces systemic inflammation and oxidative stress. In addition to systemic organ effects, the combination of factors that comprise air pollution are increasingly implicated in cognitive decline and with Alzheimer’s disease (Tonne et al., 2014; Cohen et al., 2017; Kilian and Kitazawa, 2018; Zhang X. et al., 2018; Kulick et al., 2020; Younan et al., 2021). However, a significant gap remains in identification of mechanisms underlying neurotoxicity of both acute and chronic air pollution exposures (Costa et al., 2019; Kulick et al., 2020; Schikowski and Altuğ, 2020; Gao et al., 2021). The unlikely occurrence of direct entry of particles into the brain raises consideration of the contribution from systemic effects such as alterations in respiratory function, decreased oxygen delivery to the brain, lung based pain signals to the brain, and other systemic effects of inflammatory or organ damage. Thus, this emphasizes the complex interaction of effects that occur across multiple organ systems.

Exposure to heavy metals can induce systemic inflammation, oxidative stress, and contribute to nervous system damage. They also show significant potential to alter aging biology (Jan et al., 2015; Milnerowicz et al., 2015; Xiao et al., 2021). Heavy metals often occur in mixtures in both air and drinking water, including arsenic (As), cadmium (Cd), lead (Pb), mercury (Hg), and uranium (U), with high disparity in exposure affecting the most socially vulnerable populations (Davis et al., 2016; Domingo-Relloso et al., 2019a,b; Lucchini et al., 2019; Xiao et al., 2021). While also potentially contributed to adverse outcomes, trace metals such as chromium (Cr), cobalt (Co), copper (Cu), magnesium (Mg), and zinc (Zn) found naturally and in dietary sources, may be “essential” and play important roles in maintaining multi-system homeostasis. An imbalance of essential metals can occur through dietary deficiency or exposure to other toxicants leading to dysregulation of cellular and molecular signaling necessary for normal cellular function. Individually, and as mixtures, heavy metal exposures induce oxidative stress through generation of numerous reactive oxygen species (ROS) linked to metabolic changes and free radical modification of proteins, while insufficient essential metals can alter homeostasis of the immune system resulting in systemic inflammation (Milnerowicz et al., 2015). At environmentally relevant levels, changes in inflammation and ROS underly associations of metals with numerous aging related diseases, including cardiometabolic disease (Moon et al., 2012; James et al., 2013; Tellez-Plaza et al., 2013; James et al., 2015; Lentini et al., 2017), cancer (Hayes, 1997; Nawrot et al., 2006; National Research Council, 2013), osteoporosis (James and Meliker, 2013), neurological and neurocognitive decline (Power et al., 2014). Autophagy, a mechanism underlying several complex neurodegenerative diseases, is associated with exposure to several metals and metal-induced neurotoxicity (Bakulski et al., 2020).

### Genomic Instability

Epigenetic modifications are important targets vulnerable to the impacts of exposure to environmental chemicals, with important implications for aging biology (Tsamou et al., 2018). Nonetheless, this field is in its infancy as the majority of work to date within populations has focused on DNA methylation markers. Epidemiologic studies of air pollution find epigenetic regulation of t-cells, which are important elements of the immune response (Zhong et al., 2017). Other elements of controlling gene expression and regulation, including microRNA, histone modification, and chromatin splicing may also be influenced by external factors (Bayarsaihan, 2011; Jardim, 2011; Luco et al., 2011). Arsenic and lead, for example, induce genetic and epigenetic alternations by decreasing transcription enzymes and disruption of epigenetic control (Zawia, 2003; Nawrot et al., 2006; Eid and Zawia, 2016). Both metals and air pollution exposure have been associated with accelerated biological aging in different population studies (Nwanaji-Enwerem et al., 2016; Zhong et al., 2017; White et al., 2019; Nwanaji-Enwerem et al., 2020; Nwanaji-Enwerem et al., 2021b).

Genetic instability also includes shortened telomeres and cellular metabolism. Recent work in Bangladesh shows associations with telomere length (Zhang C. et al., 2018) and suggests that genetics interact with arsenic metabolism and toxicity *via* an epigenetic mechanism (Pierce et al., 2019). Cadmium exposure has been associated with decreased leukocyte telomere length in the NHANES adult population (Zota et al., 2015). Cadmium is a toxic metal with a very long biological half-life (up to 40 years) (Diamond et al., 2003) and as the body burden of cadmium increases with age, zinc deficiency increases due to interference with absorption and transport into cells and distortion of metalloenzymes in cells, thus, promoting the aging process (Garfinkel, 1986).

## Biological Susceptibility and Social Vulnerability to Environmental Toxicants

Gene-by-environment interactions may play a substantial role in shaping the aging trajectory and risk of adverse aging-related outcomes by altering biological susceptibility to subsequent environmental chemicals. Mapping the human genome has provided tremendous insight into the heritability of disease, particularly aging-related disease, but it is clear that genetics alone are only one element of the larger disease risk equation. As an example, mutations in the WDR45 gene increase endoplasmic reticulum stress, impair control and alter organelle autophagy linked with early neurological and Parkinson’s disease in young children and adolescents. These negative processes are exacerbated by metal ions suggesting potential for gene by environment interactions underlying neurological disease pathology in older adults (Haack et al., 2013; Wan et al., 2020). Genetic mutations alter toxicity pathways making some individuals less likely to detoxify and recover from environmental exposures.

Beyond genetics, there are multiple windows of biological susceptibility during which exposure to environmental pollutants have greater impact on compensatory mechanisms than others, again altering the shape of the aging trajectory within and across generations (Swanson et al., 2009; Olvera Alvarez et al., 2018). Biological perturbations during pre- and post-natal periods, and even later in adolescence, can influence health and disease trajectories, including rate of decline (Swanson et al., 2009; Wright, 2017; Finch and Morgan, 2020). Epigenetic changes and telomere length, two well-established biomarkers of aging, have been altered *in utero* from environmental exposures including air pollution, metals, and endocrine disruptors (Kohanski et al., 2016; Cardenas et al., 2017; Lee et al., 2020), resulting in intergenerational effects (Brieno-Enriquez et al., 2015; Martos et al., 2015; Nawrot et al., 2018; Ladd-Acosta et al., 2019; Peng et al., 2019; Smith et al., 2020). Early-life exposure impacts on aging biomarkers and understanding which alterations are malleable to interventions over the life-course are important areas for future research and policy development.

Given the demonstration of early life impacts of environmental stressors on physiological systems and healthy aging, our attention must also focus on the societal (including economic factors) that conspire to exacerbate adverse outcomes overtime. Health disparities, defined as “a health difference that adversely affects defined disadvantaged populations, based on one or more health outcomes,” are generally considered preventable and unjust (Duran et al., 2019). This definition highlights the sources of disparities in aging research are largely environmental versus innate or genetic. A better integration of the broadly defined complex of environmental factors—that may contribute to excessive and premature aging would contribute to understanding the independent and collective impact of these modifiable factors on health outcomes overall and in terms of disparities. The intersections between environmental toxic exposures and social determinants, and their influence on biological markers of aging, may be key drivers in aging-related health outcomes, differential life expectancy, and quality of life.

Another aspect of aging contributing to disparities in aging outcomes and premature mortality are inherent structural factors that shape where people live as they age. Life expectancy research shows disparities across urban and rural continuums and by social gradient in the United States pointing to an important role for environmental factors. Socially vulnerable or disadvantaged older adult populations, including racial and ethnic minorities, tend to age in place (and likely across the life-course and for generations) but little research exists to document the effects of structural factors on aging or age-related disease (Clarke and Nieuwenhuijsen, 2009; Michael and Yen, 2014; Vanleerberghe et al., 2017). Disadvantaged older adults are more likely to be exposed to environmental toxicants and physical hazards than older adults with resources and assets (Michael and Yen, 2014; Vanleerberghe et al., 2017). Disadvantaged older adults also face a greater likelihood of other psychological stressors include financial strain, insufficient mental stimulation, fewer opportunities for social engagement, less access to material and social resources (e.g., senior centers), healthcare services, safety, and adequate transportation (Clarke and Nieuwenhuijsen, 2009; Won et al., 2016; Wu and Tseng, 2018; Ahn et al., 2020; Black and Jester, 2020). Disadvantage across the life-course, including early childhood has been associated with accelerated aging, including increased risk of chronic disease from which vulnerability contributes to increased biological susceptibility and reduced resilience (Marini et al., 2020).

Demographers and social scientists have focused on how shared experiences across the life course contributes to biological weathering, which is similar to frailty as a composite of chronic conditions (Krieger, 2005; Geronimus et al., 2006). Biological weathering and frailty are similar in concept to allostatic load, as an indicator of biological aging where adverse experiences can lead to stress and ultimately affect the ability of the body to maintain “allostasis” or physiological stability despite environmental challenges (Forde et al., 2019; Shiels et al., 2019; Guidi et al., 2021). Allostatic load is a maladaptive process referring to the gradual dysregulation between the autonomic nervous system, the hypothalamic pituitary axis, and the metabolic, cardiovascular, and immune systems in response to physical or psychological stress hindering the body’s ability to maintain “allostasis” or to maintain homeostasis while responding to accurate stressors or challenges (Geronimus, 2001). The effect of neighborhood as a component of environmental factors has been associated with shortening of telomeres (Park et al., 2015; Gebreab et al., 2016; Alexeeff et al., 2019). Neighborhood factors have also been associated with epigenetic predictors of all-cause mortality (Ward-Caviness et al., 2020a) and with accelerated epigenetic age (Martin et al., 2021). Accelerated epigenetic aging has also been associated with traffic-related air pollution (Ward-Caviness et al., 2020b) and fine particulate matter (PM2.5) as well as with organochlorine pesticides (Lind et al., 2018).

It is well established that living in disadvantaged neighborhoods can lead to more exposure to environmental hazards (Morello-Frosch et al., 2011; Mascarenhas et al., 2021). These environmental hazards also often coincide with other detrimental social and economic factors including lower income households, poorer housing and higher social stress (Morello-Frosch and Jesdale, 2006; Mohai et al., 2009; Morello-Frosch et al., 2011). In the United States, historical racism, including red-lining and other social stratification limited options for where Black and other communities of color could purchase or rent housing. Communities deemed less than ideal were also located in close proximity to industrial factories, factories, hazardous waste sites, and less than desirable lands at the same time that these same communities were struggling to obtain equal job opportunities (Bullard et al., 2008). Thus, many lower income communities of color experience increased toxicant exposures and social stressors (Huang and Barzyk, 2017). Extensive environmental justice research shows environmental toxicant exposures are highest among non-white and lower income communities in the United States (Mohai et al., 2009; Morello-Frosch et al., 2011; Mohai and Saha, 2015). These communities are also those with limited access to resources and assets such as healthy food outlets, high quality healthcare, green space and other opportunities for recreation and physical activity. Recent studies have used hierarchical clustering methods to show that neighborhood socioeconomic and sociodemographic factors characterizing disadvantaged communities are associated with adverse cardiovascular and metabolic outcomes (Mirowsky et al., 2017; Weaver et al., 2022) and that exposure to fine particulate matter (PM2.5) exacerbates this in a neighborhood cluster characterized as urban, low socioeconomic status, and non-white (Weaver et al., 2019). Among a general population-based sample, individuals who felt their neighborhoods were poorly maintained, experienced stress from crime, or reported their neighborhood as not well maintained were found to have an lower lung function (FEV 1) associate with chronic PM 2.5 exposure at with exposures less than 10 ug/m3 PM 2.5, the World Health Organization health based standard prior to 202 (Malecki et al., 2018). Neighborhood psychosocial hazards together with lead exposure have also been shown to be associated with persistent cognitive decline in older adults (Glass et al., 2009). Evidence is growing to support the need to include both chemical toxicant and non-chemical stress exposure in environment and aging research (McEwen and Tucker, 2011; Gaston and Jackson, 2021; Jackson et al., 2021; Nwanaji-Enwerem et al., 2021a).

## Integrating the Science of Aging and Environmental Health Research

Technological advances have opened up new avenues of molecular and biological research to better identify key hallmarks of aging (Peters et al., 2021). These hallmarks represent overlapping biological mechanisms that shape the aging trajectory. Technology provides increasingly more accessible and cost-effective analyses, allowing more multi-omic platforms to be used in toxicology and molecular epidemiology. With these advances, integrating environmental health and aging biology becomes more intriguing and opens new avenues for transformational research.

As the underpinnings of aging biology continue to emerge, there are numerous opportunities to advance our understanding of how aging phenotypes are shaped by environmental factors—broadly speaking—and more specifically with respect to environmental toxicants. The aging-related alterations in the genetic and physiological systems and co-morbidities discussed throughout this paper highlight the reduced capacity for the biological compensatory mechanisms in older adults to maintain homeostasis in the face of exposure to external stressors. Research at the intersection of aging and the environment has the opportunity to inform important knowledge gaps regarding malleable features of aging and inform prevention and policies to protect population health and address health equity. The integration of these fields can fill critical gaps in identification of modifiable risk factors, opportunities for new therapeutic interventions, and support for new policies and programs. Critical new approaches and pathways for understanding environmental health that can be integrated with aging research to address many knowledge gaps and shape future policy directions.

### The Exposome Framework in Environment—Cumulative Exposures, Systems Biology, and Aging Research

Considering the substantial contribution of external environmental factors on aging biology compared to genetics, new research is needed to examine the intersection of environmental toxins with key elements of aging biology. The exposome concept was originally proposed by Chris Wild, in 2005, to include all exposures from conception onward, including diet, lifestyle, and environment. This concept now shapes much of new environmental health sciences research. The concept has been expanded to also explicitly acknowledge that social factors tend to shape the exposome through numerous social and other determinants. In the United States, for example, historical racism and widening wealth gap have left many disadvantaged communities sharing an unequal burden of environmental exposures, and these communities face additional mental health, economic, and social stressors. With increased understanding that only a fraction of aging is shaped by genetics, the exposome concept supports a new framework for advancing both aging and environmental health sciences. Defined as “the cumulative measure of environmental influences and associated biological responses throughout the lifespan including exposures from the environment, diet, behavior, and endogenous processes,” the exposome approach represents an unprecedented opportunity to more comprehensively investigate both the fundamental processes of aging and aging disparities (Cifuentes et al., 2019; Juarez et al., 2020a,b; Ogojiaku et al., 2020).

The Exposome Framework highlights the value of technological advances in shaping these new approaches and to advance environment and aging research. High resolution mass-spectrometry allows for identification of exogenous and endogenous small molecules used to characterize thousands of circulating environmental chemicals and metabolites simultaneously (Vermeulen et al., 2020). Combined with additional epigenetic and microbiome and phenotypic data, the contribution of environmental mixtures on biological systems and aging biology can begin to be explored. For example, in animal models metabolomics has been used to differentiate normative aging from aging related to Alzheimer’s disease (Hunsberger et al., 2020). This same experimental design could be used to better understand the contribution of toxic chemicals in altering the normative aging metabolic profiles and impacts on the Alzheimer’s disease profiles. To date, these integrated assessments have not been conducted despite increased understanding of cellular and tissues specific metabolisms in aging biology (Zhang et al., 2021). Thus, this integrated approach can support new discoveries in aging research through identification of previously unidentified mixtures of chemicals and associations with hallmarks of aging. The integration of these transdisciplinary methods can also help identify clusters of environmental exposures in certain geographical areas and their biological impact in a manner that can inform effective interventions at multiple levels.

Expanding the biological exposome framework with a social-exposome can advance exposure characterization, incorporate new statistical methods and technologies, as well as strengthen cumulative risk models. For example, new mass-spectrometry approaches combined with next gen sequencing, questionnaire, and GIS methods can holistically characterize the broad range of environmental and social exposure pathways relevant to aging biology. The exposome approach also overcome several common study design limitations in environmental health and aging research. In particular, a lack of longitudinal cohorts among younger and middle age adults limits understanding of how aging trajectories in populations differ in time and space. The exposome approach also begins to address the historical one pollutant and one endpoint approaches to toxicology, and environmental epidemiology studies that often lack data necessary to translate findings into real-world setting (Juarez et al., 2020a). To this end, the social exposome allows researchers to better determine biological mechanisms beyond genetics by which environmental and social factors intersect to accelerate biological aging and health disparities (Nwanaji-Enwerem et al., 2021b).

### Advancing Aging and Environmental Health Sciences Research

#### Environmental Justice and Health Disparities—Integrating Social Sciences in Environment and Aging Research

Beyond biological susceptibility, population level vulnerability to chemical exposures also varies across time and place with greater potential for exposure to adverse chemical mixtures and non-chemical stressors in more disadvantaged communities (Davis et al., 2016; Lucchini et al., 2019). The interaction between the broad spectrum of factors that represent the “environment” and influence on aging beyond early development and childhood are only recently being investigated to any significant level (Carro et al., 2013). Current research and intervention approaches have not resulted in reduced disparities and have not provided sufficient information to target interventions that address the fundamental causes of disparities. Despite the growing recognition regarding the complexity of environmental determinants of health, researchers have traditionally taken an over-simplified, reductionist approach to investigate the health impacts of myriad chemical or non-chemical stressors. Until recently, each factor has been studied independently and in isolation. Even as research on toxic effects of chemical mixtures has evolved, the role that non-chemical stressors including diet and social disadvantage play in healthy aging over the life-course has been largely overlooked. Environmental justice recognizes that social and other factors shape unequal distribution of physical and chemical hazards in the environment, increasing vulnerability for adverse exposures in some communities more than others. At the same time, these disadvantaged communities face greater social stressors that shape biological aging. More holistic research that captures and comprehensively investigates the complex nature of environmental determinants of health and disparities needs to be conducted across diverse populations. Social scientists have long known that place matters to health and aging, and with the advancement of biomarker research, there is a unique opportunity to begin to integrate biology with social and environmental science to understand how and why communities and individuals age differently and what can be modified to reduce significant and persistent disparities. This research could provide the evidence-base to support key tenants of environmental justice and lead to the enforcement of environmental laws, regulations, and policies in a fair and meaningfully way across the entire population.

#### Climate, the Built Environment, and One Health

Aging must also be considered in the context of other environmental factors such as the built environment, climate change, environmental policy, programs, and interventions. Accelerated aging has also long been an indicator of population social vulnerability to climate change, as older adults are more likely to live in institutional settings and/or have lower mobility and capacity to live independently. Also, within an aged population, multiple chronic conditions and frailty make overcoming the physical hazards inherent in extreme weather events, and subsequent changes to water quality and physical environment, more challenging.

One health acknowledges that human health is closely related to shared environments and health of animals within these environments (Destoumieux-Garzón et al., 2018; Mackenzie and Jeggo, 2019). One health is closely linked to climate change as key drivers of climate change including land use changes such as deforestation, intensive farming and shifting habitat conditions increase the probability of human contact and for diseases in animals to pass to humans (Daszak et al., 2001; Hoelzer et al., 2017). Examples of diseases that have shifted overtime due to changing landscapes include Lyme disease, Salmonella, rabies, and COVID-19 (Jones et al., 2008). Other zoonotic diseases of public health concern including antimicrobial resistance, and food safety (Hoelzer et al., 2017). Most recently, the COVID-19 pandemic has highlighted increased vulnerability of older adults to infectious agents, and scholars predict future pandemics beyond COVID-19 will continue to pose significant threats to population health (Cleaveland et al., 2001; Mackenzie and Smith, 2020). Environmental factors, including changing landscapes, increasing development in diverse habitats and climate change, all key elements of “One Health” will also exacerbate risks to aging populations. Like climate change and built environment, collaborative and multidisciplinary approaches to address issues posed by one health at local, national and global levels will be needed to reduce significant risks to aging populations (Jones et al., 2008).

As the global population continues to age, both the potential acceleration of toxic effects of environmental pollutants and increasing risk of exposure to physical hazards will have important population health impacts. Alterations to urban environments, such as increasing green space, can also have important co-benefits by reducing pollution levels and boosting mental health, and offering places for older adults to maintain an active lifestyle as they age. All of these factors can support increased wellness and potentially reduce toxic effects of pollutants, but in order for these efforts to be successful, aging and environmental health scientists much come together to support this research.

### Environmental Regulations, Policy Development, and Research Translation

The Safe Drinking Water Act (SDWA), the Clean Air Act (CAA), and the Frank R Lautenberg Chemical Safety for the 21st Century (TSCA Reform) Act have existing provisions for protecting susceptible sub-populations, including older adults (Hooper and Kaufman, 2018). According to the Lautenberg Act, the term ‘potentially exposed or susceptible subpopulation’ means a group of individuals within the general population identified by either greater susceptibility or greater exposure, may be at greater risk than the general population of adverse health effects from exposure to a chemical substance or mixture. This includes infants, children, pregnant women, workers, or older adults. The SDWA and CAA include similar language with the intent to protect susceptible groups and life-stages. The United States Environmental Protection Agency’s Integrated Science Assessment for Particulate Matter includes discussion of the evidence-base for increased susceptibility associated with aging and with co-morbidities associated with aging (Environmental Protection Agency, 2019). The US Food and Drug Administration has offered interim guidance documents for consideration of older adults in clinical trials (Food and Drug Administration, 2020). Despite such regulatory acknowledgment, to advance policy and decisions that impact the population, underlying evidence at the intersection of aging biology and environmental health sciences is required. Identifying an approach to advance the integration of aging biomarkers and underlying mechanisms of action in this risk assessment process is necessary. Additional research would support renewed emphasis on aging populations as susceptible sub-populations and offer more sensitive endpoints for integration into existing regulatory decision-making. With increasing availability of big data readily accessible to environmental health scientists, including genomic, metabolomics, proteomic, and lipodomic approaches, researchers are able to more readily identify interim biomarkers of exposure and response that can identify subtle biological changes related to environmental exposures that can proceed clinical diagnosis of age-related diseases. Another challenge that systems biology approaches can overcome is the increased understanding of how low-level exposures accumulated over the life-course may also play in exacerbating the risk of age-related phenotypes and altering the process of biological aging.

Environmental risk assessment continues to evolve with increased understanding of systems biology in determining adverse effects of environmental pollutants on human health. Traditional approaches to environmental health sciences including dose-response assessment in toxicity and environmental epidemiology have long acknowledged the importance of timing of exposure and response in determining chemical toxicity. Regulatory paradigms for estimating environmental chemical toxicity have focused on individual chemicals. However, individuals are exposed to complex mixtures of both environmental and social stressors and buffers throughout their life that can increase or decrease resilience and recovery and contribute to aging trajectories.

A renewed emphasis is needed on research translation from bench to bedside and into the community. Gerontologists may not always be aware of the cumulative environmental exposures that are currently impacting clinical care and outcomes, or the cumulative exposures that have shaped the aging phenotypes of patients. In addition, the potential for interactions with polypharmacy for older adults associated with comorbidities is likely not appreciated (Ginsberg et al., 2005). Too often, the general public, policymakers, clinicians, and researchers in domains outside of environmental health do not consider the important role that environmental chemical and non-chemical stressors play in shaping aging biology and longevity. Primary prevention to reduce exposures would reduce the contribution of environmental stressors on the aging trajectory and minimize the adverse effects of chemical stressors on individuals as they age. Advancing environmental health literacy will also increase support for primary prevention strategies, including reducing environmental exposures among the Nation’s most vulnerable populations.

## Conclusion

To achieve these policy and research goals, a new multi-agency perspective is needed, with increased attention toward team science and gerotoxicology research. Integrating aging biology and environmental health sciences will lead to transformational research, better policy development, and better clinical outcomes. The toxicity of environmental chemicals in older adulthood also brings health disparities in longevity and health status among older adults into sharper focus (Nwanaji-Enwerem et al., 2021b). Aging alters the fundamental pharmacokinetic and pharmacodynamic processes of chemicals, and research is needed to examine how these drivers of biological susceptibility intersect with social vulnerability (Geller and Zenick, 2005). Increased understanding may support new and alternative treatments that consider patient metabolism and toxicity in older aged adults and provide the basis for effective regulatory activity. Reducing toxic exposures in older adulthood may also extend quality of life and opportunities to remain disease free.

## Author Contributions

All authors contributed equally to manuscript conceptualization, added contributions to the text, contributed to the article, and approved the submitted version. KM facilitated manuscript development. GH, KJ, JA, and MO provided significant edits and equally contributed to manuscript development with KM. GH provided significant final editing and led agency review of the final manuscript. AG contributed to additional content post review. AG, CJ, and GM contributed to components and final editing.

## Conflict of Interest

The authors declare that the research was conducted in the absence of any commercial or financial relationships that could be construed as a potential conflict of interest.

## Publisher’s Note

All claims expressed in this article are solely those of the authors and do not necessarily represent those of their affiliated organizations, or those of the publisher, the editors and the reviewers. Any product that may be evaluated in this article, or claim that may be made by its manufacturer, is not guaranteed or endorsed by the publisher.

## References

[B1] Agache I. Miller R. Gern J. E. Hellings P. W. Jutel M. Muraro A. (2019). Emerging concepts and challenges in implementing the exposome paradigm in allergic diseases and asthma: a Practall document. *Allergy.* 74 449–463. 10.1111/all.13690 30515837

[B2] Ahadi S. Zhou W. Schüssler-Fiorenza Rose S. M. Sailani M. R. Contrepois K. Avina M. (2020). Personal aging markers and ageotypes revealed by deep longitudinal profiling. *Nat. Med.* 26 83–90. 10.1038/s41591-019-0719-5 31932806 PMC7301912

[B3] Ahn M. Kwon H. J. Kang J. (2020). Supporting aging-in-place well: findings from a cluster analysis of the reasons for aging-in-place and perceptions of well-being. *J. Appl. Gerontol.* 39 3–15. 10.1177/0733464817748779 29277156

[B4] Alexeeff S. E. Schaefer C. A. Kvale M. N. Shan J. Blackburn E. H. Risch N. (2019). Telomere length and socioeconomic status at neighborhood and individual levels among 80,000 adults in the genetic epidemiology research on adult health and aging cohort. *Environ. Epidemiol.* 3:3. 10.1097/EE9.0000000000000049 33778338 PMC7939422

[B5] Apóstolo J. Cooke R. Bobrowicz-Campos E. Santana S. Marcucci M. Cano A. (2017). Predicting risk and outcomes for frail older adults: an umbrella review of frailty screening tools. *JBI Datab. Syst. Rev. Impl. Rep.* 15 1154–1208. 10.11124/JBISRIR-2016-003018 PMC5457829 28398987

[B6] Austad S. N. Hoffman J. M. (2018). Is antagonistic pleiotropy ubiquitous in aging biology? *Evol. Med. Public Health* 2018 287–294. 10.1093/emph/eoy033 30524730 PMC6276058

[B7] Bakulski K. M. Seo Y. A. Hickman R. C. Brandt D. Vadari H. S. Hu H. (2020). Heavy metals exposure and alzheimer’s disease and related dementias. *J. Alzheimers Dis.* 76 1215–1242. 10.3233/JAD-200282 32651318 PMC7454042

[B8] Barker D. J. Winter P. D. Osmond C. Margetts B. Simmonds S. J. (1989). Weight in infancy and death from ischaemic heart disease. *Lancet* 2 577–580. 2570282 10.1016/s0140-6736(89)90710-1

[B9] Bayarsaihan D. (2011). Epigenetic mechanisms in inflammation. *J. Dent. Res.* 90 9–17. 10.1177/0022034510378683 21178119 PMC3144097

[B10] Bektas A. Schurman S. H. Sen R. Ferrucci L. (2018). Aging, inflammation and the environment. *Exp. Gerontol.* 105 10–18. 10.1016/j.exger.2017.12.015 29275161 PMC5909704

[B11] Belsky D. W. Moffitt T. E. Cohen A. A. Corcoran D. L. Levine M. E. Prinz J. A. (2018). Eleven telomere, epigenetic clock, and biomarker-composite quantifications of biological aging: do they measure the same thing? *Am. J. Epidemiol.* 187 1220–1230. 10.1093/aje/kwx346 29149257 PMC6248475

[B12] Ben-Shlomo Y. Cooper R. Kuh D. (2016). The last two decades of life course epidemiology, and its relevance for research on ageing. *Int. J. Epidemiol.* 45 973–988. 10.1093/ije/dyw096 27880685 PMC5841628

[B13] Bernardes de Jesus B. Blasco M. A. (2012). Assessing cell and organ senescence biomarkers. *Circul. Res.* 111 97–109. 10.1161/CIRCRESAHA.111.247866 22723221 PMC4824275

[B14] Black K. Jester D. J. (2020). Examining older adults’ perspectives on the built environment and correlates of healthy aging in an american age-friendly community. *Int. J. Environ. Res. Public Health* 17:7056. 10.3390/ijerph17197056 32992480 PMC7578930

[B15] Bliwise D. L. Ansari F. P. Straight L. B. Parker K. P. (2005). Age changes in timing and 24-hour distribution of self-reported sleep. *Am. J. Geriatr. Psychiatry.* 13 1077–1082. 10.1176/appi.ajgp.13.12.1077 16319300

[B16] Bortz W. M. II (2002). A conceptual framework of frailty: a review. *J. Gerontol. A Biol. Sci. Med. Sci.* 57 M283–M288. 10.1093/gerona/57.5.m283 11983721

[B17] Bosquet Enlow M. Sideridis G. Bollati V. Hoxha M. Hacker M. R. Wright R. J. (2019). Maternal cortisol output in pregnancy and newborn telomere length: Evidence for sex-specific effects. *Psychoneuroendocrinology* 102 225–235. 10.1016/j.psyneuen.2018.12.222 30590340 PMC6420355

[B18] Bourdrel T. Annesi-Maesano I. Alahmad B. Maesano C. N. Bind M.-A. (2021). The impact of outdoor air pollution on COVID-19: a review of evidence from animal, and human studies. *Eur. Respirat. Rev.* 30:200242. 10.1183/16000617.0242-2020 33568525 PMC7879496

[B19] Brieno-Enriquez M. A. Garcia-Lopez J. Cardenas D. B. Guibert S. Cleroux E. Ded L. (2015). Exposure to endocrine disruptor induces transgenerational epigenetic deregulation of microRNAs in primordial germ cells. *PLoS One* 10:e0124296. 10.1371/journal.pone.0124296 25897752 PMC4405367

[B20] Brugha R. Grigg J. (2014). Urban air pollution and respiratory infections. *Paediatr. Respir. Rev.* 15 194–199. 10.1016/j.prrv.2014.03.001 24704510

[B21] Brunekreef B. Holgate S. T. (2002). Air pollution and health. *Lancet* 360 1233–1242. 12401268 10.1016/S0140-6736(02)11274-8

[B22] Bullard R. D. Mohai P. Saha R. K. Wright B. H. (2008). Toxic wastes and race at twenty: why race still matters after all of these years. *Environ. Law* 38:371.

[B23] Byars S. G. Voskarides K. (2020). Antagonistic pleiotropy in human disease. *J. Mol. Evol.* 88 12–25. 10.1007/s00239-019-09923-2 31863128

[B24] Cardenas A. Rifas-Shiman S. L. Agha G. Hivert M.-F. Litonjua A. A. DeMeo D. L. (2017). Persistent DNA methylation changes associated with prenatal mercury exposure and cognitive performance during childhood. *Sci. Rep.* 7:288. 10.1038/s41598-017-00384-5 28325913 PMC5428306

[B25] Carro T. Dean K. Ottinger M. A. (2013). Effects of an environmentally relevant polychlorinated biphenyl (PCB) mixture on embryonic survival and cardiac development in the domestic chicken. *Environ. Toxicol. Chem.* 32 1325–1331. 10.1002/etc.2178 23418095

[B26] Castro-Mejía J. L. Khakimov B. Krych L. Bulow J. Bechshoft R. L. Hojfeldt G. (2020). Physical fitness in community-dwelling older adults is linked to dietary intake, gut microbiota, and metabolomic signatures. *Aging Cell.* 19 e13105. 10.1111/acel.13105 31967716 PMC7059135

[B27] Cheung A. C. Walker D. I. Juran B. D. Miller G. W. Lazaridis K. N. (2020). Studying the exposome to understand the environmental determinants of complex liver diseases. *Hepatology* 71 352–362. 10.1002/hep.31028 31701542 PMC7329010

[B28] Cifuentes P. Reichard J. Im W. Smith S. Colen C. Giurgescu C. (2019). Application of the public health exposome framework to estimate phenotypes of resilience in a model ohio african-american women’s cohort. *J. Urban Health* 96 (Suppl. 1), 57–71. 10.1007/s11524-018-00338-w 30758792 PMC6430281

[B29] Clarke P. Nieuwenhuijsen E. R. (2009). Environments for healthy ageing: A critical review. *Maturitas* 64 14–19. 10.1016/j.maturitas.2009.07.011 19695800

[B30] Cleaveland S. Laurenson M. K. Taylor L. H. (2001). Diseases of humans and their domestic mammals: pathogen characteristics, host range and the risk of emergence. *Philos. Trans. R Soc. Lond. B Biol. Sci.* 356 991–999. 10.1098/rstb.2001.0889 11516377 PMC1088494

[B31] Cohen A. J. Brauer M. Burnett R. Anderson H. R. Frostad J. Estep K. (2017). Estimates and 25-year trends of the global burden of disease attributable to ambient air pollution: an analysis of data from the Global Burden of Diseases Study 2015. *Lancet* 389 1907–1918. 10.1016/S0140-6736(17)30505-6 28408086 PMC5439030

[B32] Colicino E. Cowell W. Bozack A. Foppa Pedretti N. Joshi A. Niedzwiecki M. M. (2020). Association between prenatal immune phenotyping and cord blood leukocyte telomere length in the PRISM pregnancy cohort. *Environ. Res.* 191:110113. 10.1016/j.envres.2020.110113 32841635 PMC7883408

[B33] Collins A. R. (2014). Measuring oxidative damage to DNA and its repair with the comet assay. *Biochim. Biophys. Acta.* 1840 794–800. 10.1016/j.bbagen.2013.04.022 23618695

[B34] Conway J. (2021). Ageing of the gut microbiome: Potential influences on immune senescence and inflammageing. *Ageing Res. Rev.* 68:101323. 10.1016/j.arr.2021.101323 33771720

[B35] Costa L. G. Cole T. B. Dao K. Chang Y.-C. Garrick J. M. (2019). Developmental impact of air pollution on brain function. *Neurochem. Int.* 131:104580. 10.1016/j.neuint.2019.104580 31626830 PMC6892600

[B36] Costantini E. D’Angelo C. Reale M. (2018). The Role of immunosenescence in neurodegenerative diseases. *Mediat. Inflam.* 2018:6039171. 10.1155/2018/6039171 PMC5863336 29706800

[B37] Cowell W. Colicino E. Tanner E. Amarasiriwardena C. Andra S. S. Bollati V. (2020). Prenatal toxic metal mixture exposure and newborn telomere length: Modification by maternal antioxidant intake. *Environ. Res.* 190:110009. 10.1016/j.envres.2020.110009 32777275 PMC7530067

[B38] Craven H. McGuinness D. Buchanan S. Galbraith N. McGuinness D. H. Jones B. (2021). Socioeconomic position links circulatory microbiota differences with biological age. *Sci. Rep.* 11:12629. 10.1038/s41598-021-92042-0 34135381 PMC8209159

[B39] Crome P. (2003). What’s different about older people. *Toxicology* 192 49–54. 10.1016/s0300-483x(03)00253-1 14511903

[B40] Cryan J. F. O’Riordan K. J. Sandhu K. Peterson V. Dinan T. G. (2020). The gut microbiome in neurological disorders. *Lancet Neurol.* 19 179–194. 31753762 10.1016/S1474-4422(19)30356-4

[B41] Cuervo A. M. Macian F. (2014). Autophagy and the immune function in aging. *Curr. Opin. Immunol.* 29 97–104. 10.1016/j.coi.2014.05.006 24929664 PMC4119564

[B42] Cuervo A. M. Bergamini E. Brunk U. T. Droge W. Ffrench M. Terman A. (2005). Autophagy and aging: the importance of maintaining “clean” cells. *Autophagy* 1 131–140. 10.4161/auto.1.3.2017 16874025

[B43] Daszak P. Cunningham A. A. Hyatt A. D. (2001). Anthropogenic environmental change and the emergence of infectious diseases in wildlife. *Acta Trop.* 78 103–116. 10.1016/s0001-706x(00)00179-0 11230820

[B44] Davis H. T. Aelion C. M. Liu J. Burch J. B. Cai B. Lawson A. B. (2016). Potential sources and racial disparities in the residential distribution of soil arsenic and lead among pregnant women. *Sci. Total. Environ.* 55 622–630. 10.1016/j.scitotenv.2016.02.018 26897405 PMC4808624

[B45] Destoumieux-Garzón D. Mavingui P. Boetsch G. Boissier J. Darriet F. Duboz P. (2018). The one health concept: 10 years old and a long road ahead. *Front. Vet. Sci.* 5:14. 10.3389/fvets.2018.00014 29484301 PMC5816263

[B46] Dhingra R. Nwanaji-Enwerem J. C. Samet M. Ward-Caviness C. K. D. N. A. (2018). Methylation age-environmental influences, health impacts, and its role in environmental epidemiology. *Curr. Environ. Health Rep.* 5 317–327. 10.1007/s40572-018-0203-2 30047075 PMC6173330

[B47] Diamond G. L. Thayer W. C. Choudhury H. (2003). Pharmacokinetics/pharmacodynamics (PK/PD) modeling of risks of kidney toxicity from exposure to cadmium: estimates of dietary risks in the U.S. population. *J. Toxicol. Environ. Health* 66 2141–2164. 10.1080/15287390390227589 14710597

[B48] Doiron D. de Hoogh K. Probst-Hensch N. Fortier I. Cai Y. De Matteis S. (2019). Air pollution, lung function and COPD: results from the population-based UK Biobank study. *Eur. Respirat. J.* 54:1802140. 10.1183/13993003.02140-2018 31285306

[B49] Domingo-Relloso A. Grau-Perez M. Briongos-Figuero L. Gomez-Ariza J. L. Garcia-Barrera T. Duenas-Laita A. (2019a). The association of urine metals and metal mixtures with cardiovascular incidence in an adult population from Spain: the Hortega Follow-Up Study. *Int. J. Epidemiol.* 48 1839–1849. 10.1093/ije/dyz061 31329884 PMC6929535

[B50] Domingo-Relloso A. Grau-Perez M. Galan-Chilet I. Garrido-Martinez M. J. Tormos C. Navas-Acien A. (2019b). Urinary metals and metal mixtures and oxidative stress biomarkers in an adult population from Spain: The Hortega Study. *Environ. Int.* 123 171–180. 10.1016/j.envint.2018.11.055 30529889

[B51] Duran D. Asada Y. Millum J. Gezmu M. (2019). Harmonizing health disparities measurement. *Am. J. Public Health* 109:S25. 10.2105/AJPH.2019.304952 30699026 PMC6356133

[B52] Eid A. Zawia N. (2016). Consequences of lead exposure, and it’s emerging role as an epigenetic modifier in the aging brain. *Neurotoxicology* 56 254–261. 10.1016/j.neuro.2016.04.006 27066759

[B53] Elfawy H. A. Das B. (2019). Crosstalk between mitochondrial dysfunction, oxidative stress, and age related neurodegenerative disease: Etiologies and therapeutic strategies. *Life Sci.* 218 165–184. 10.1016/j.lfs.2018.12.029 30578866

[B54] Elliott M. L. Caspi A. Houts R. M. Ambler A. Broadbent J. M. Hancox R. J. (2021). Disparities in the pace of biological aging among midlife adults of the same chronological age have implications for future frailty risk and policy. *Nat. Aging* 1 295–308. 10.1038/s43587-021-00044-4 33796868 PMC8009092

[B55] Environmental Protection Agency (2019). *Integrated Science Assessment (ISA) for Particulate Matter.* Washington, DC: EPA. 36630543

[B56] Ferraro K. F. Kemp B. R. Williams M. M. (2017). Diverse aging and health inequality by race and ethnicity. *Innov. Aging* 1:igx002. 10.1093/geroni/igx002 29795805 PMC5954610

[B57] Ferrucci L. Fabbri E. (2018). Inflammageing: chronic inflammation in ageing, cardiovascular disease, and frailty. *Nat. Rev. Cardiol.* 15 505–522. 10.1038/s41569-018-0064-2 30065258 PMC6146930

[B58] Ferrucci L. Gonzalez-Freire M. Fabbri E. Simonsick E. Tanaka T. Moore Z. (2020). Measuring biological aging in humans: A quest. *Aging Cell* 19:e13080. 10.1111/acel.13080 31833194 PMC6996955

[B59] Finch C. E. Morgan T. E. (2020). Developmental exposure to air pollution, cigarettes, and lead: implications for brain aging. *Ann. Rev. Dev. Psychol.* 2 585–614.

[B60] Food and Drug Administration (2020). *Inclusion of Older Adults in Cancer Clinical Trials Draft Guidance for Industry.* Rockville, MD: FDA.

[B61] Forde A. T. Crookes D. M. Suglia S. F. Demmer R. T. (2019). The weathering hypothesis as an explanation for racial disparities in health: a systematic review. *Ann. Epidemiol.* 33 1–18. 10.1016/j.annepidem.2019.02.011 30987864 PMC10676285

[B62] Franceschi C. Campisi J. (2014). Chronic inflammation (inflammaging) and its potential contribution to age-associated diseases. *J. Gerontol. A Biol. Sci. Med. Sci.* 69 (Suppl. 1), S4–S9. 10.1093/gerona/glu057 24833586

[B63] Franceschi C. Garagnani P. Morsiani C. Conte M. Santoro A. Grignolio A. (2018). The continuum of aging and age-related diseases: common mechanisms but different rates. *Front. Med.* 5:61. 10.3389/fmed.2018.00061 29662881 PMC5890129

[B64] Fried L. P. Tangen C. M. Walston J. Newman A. B. Hirsch C. Gottdiener J. (2001). Frailty in older adults: evidence for a phenotype. *J. Gerontol. A Biol. Sci. Med. Sci.* 56 M146–M156. 11253156 10.1093/gerona/56.3.m146

[B65] Fulop T. Larbi A. Dupuis G. Le Page A. Frost E. H. Cohen A. A. (2017). Immunosenescence and inflamm-aging as two sides of the same coin: friends or foes? *Front. Immunol.* 8:1960. 10.3389/fimmu.2017.01960 29375577 PMC5767595

[B66] Furman D. Campisi J. Verdin E. Carrera-Bastos P. Targ S. Franceschi C. (2019). Chronic inflammation in the etiology of disease across the life span. *Nat. Med.* 25 1822–1832. 10.1038/s41591-019-0675-0 31806905 PMC7147972

[B67] Gao X. Colicino E. Shen J. Just A. C. Nwanaji-Enwerem J. C. Wang C. (2018). Accelerated DNA methylation age and the use of antihypertensive medication among older adults. *Aging* 10 3210–3228. 30414594 10.18632/aging.101626 PMC6286862

[B68] Gao X. Coull B. Lin X. Vokonas P. Spiro A. Hou L. (2021). Short-term air pollution, cognitive performance and nonsteroidal anti-inflammatory drug use in the Veterans Affairs Normative Aging Study. *Nat. Aging* 1 430–437. 10.1038/s43587-021-00060-4 34841262 PMC8622756

[B69] Gareau M. G. (2016). “Chapter Eleven - Cognitive Function and the Microbiome,” in *International Review of Neurobiology*, eds Cryan J. F. Clarke G. (Cambridge: Academic Press), 227–246. 10.1016/bs.irn.2016.08.001 27793221

[B70] Garfinkel D. (1986). Is aging inevitable? The intracellular zinc deficiency hypothesis of aging. *Med. Hypoth.* 19 117–137. 10.1016/0306-9877(86)90053-8 3517602

[B71] Gaston S. A. Jackson C. L. (2021). Strengthening the case for early-life interventions to address racial/ethnic sleep disparities across the life-course using an exposome approach. *Sleep* 44:11. 10.1093/sleep/zsab182 34272566 PMC8598170

[B72] Gebreab S. Y. Riestra P. Gaye A. Khan R. J. Xu R. Davis A. R. (2016). Perceived neighborhood problems are associated with shorter telomere length in African American women. *Psychoneuroendocrinology* 69 90–97. 10.1016/j.psyneuen.2016.03.018 27070760 PMC5051547

[B73] Geller A. M. Zenick H. (2005). Aging and the environment: a research framework. *Environ. Health Perspect.* 113 1257–1262. 10.1289/ehp.7569 16140638 PMC1280412

[B74] Gensous N. Bacalini M. G. Pirazzini C. Marasco E. Giuliani C. Ravaioli F. (2017). The epigenetic landscape of age-related diseases: the geroscience perspective. *Biogerontology* 18 549–559. 10.1007/s10522-017-9695-7 28352958 PMC5514215

[B75] Geronimus A. T. (2001). Understanding and eliminating racial inequalities in women’s health in the United States: the role of the weathering conceptual framework. *J. Am. Med. Womens Assoc.* 56 49–50. 11759779

[B76] Geronimus A. T. Hicken M. T. Pearson J. A. Seashols S. J. Brown K. L. Cruz T. D. (2010). Do US black women experience stress-related accelerated biological aging?: a novel theory and first population-based test of black-white differences in telomere length. *Hum. Nat.* 21 19–38. 10.1007/s12110-010-9078-0 20436780 PMC2861506

[B77] Geronimus A. T. Hicken M. Keene D. Bound J. (2006). “Weathering” and age patterns of allostatic load scores among blacks and whites in the United States. *Am. J. Public Health* 96 826–833. 10.2105/AJPH.2004.060749 16380565 PMC1470581

[B78] Gilbert J. A. Blaser M. J. Caporaso J. G. Jansson J. K. Lynch S. V. Knight R. (2018). Current understanding of the human microbiome. *Nat. Med.* 24 392–400. 10.1038/nm.4517 29634682 PMC7043356

[B79] Ginsberg G. Hattis D. Russ A. Sonawane B. (2005). Pharmacokinetic and pharmacodynamic factors that can affect sensitivity to neurotoxic sequelae in elderly individuals. *Environ. Health Perspect.* 113 1243–1249. 10.1289/ehp.7568 16140636 PMC1280410

[B80] Glass T. A. Bandeen-Roche K. McAtee M. Bolla K. Todd A. C. Schwartz B. S. (2009). Neighborhood psychosocial hazards and the association of cumulative lead dose with cognitive function in older adults. *Am. J. Epidemiol.* 169 683–692. 10.1093/aje/kwn390 19155330 PMC2727211

[B81] Gorham S. L. Ottinger M. A. (1986). Sertoli cell tumors in Japanese quail. *Avian Dis.* 30 337–339. 10.2307/1590537 3729877

[B82] Guarnieri T. Abruzzo P. M. Bolotta A. (2020). More than a cell biosensor: aryl hydrocarbon receptor at the intersection of physiology and inflammation. *Am. J. Physiol. Cell Physiol.* 318 C1078–C1082. 10.1152/ajpcell.00493.2019 32208988

[B83] Guerville F. De Souto Barreto P. Ader I. Andrieu S. Casteilla L. Dray C. (2020). Revisiting the hallmarks of aging to identify markers of biological age. *J. Prev. Alzheimers Dis.* 7 56–64. 10.14283/jpad.2019.50 32010927

[B84] Guidi J. Lucente M. Sonino N. Fava G. A. (2021). Allostatic load and its impact on health: a systematic review. *Psychother. Psychos.* 90 11–27. 10.1159/000510696 32799204

[B85] Gundert-Remy U. Damm G. Foth H. Freyberger A. Gebel T. Golka K. (2015). High exposure to inorganic arsenic by food: the need for risk reduction. *Arch. Toxicol.* 89 2219–2227. 10.1007/s00204-015-1627-1 26586021

[B86] Haack T. B. Hogarth P. Gregory A. Prokisch H. Hayflick S. J. (2013). “Chapter Four - BPAN: The Only X-Linked Dominant NBIA Disorder,” in *International Review of Neurobiology*, eds Bhatia K. P. Schneider S. A. (Cambridge: Academic Press), 85–90. 10.1016/B978-0-12-410502-7.00005-3 24209435

[B87] Hannum G. Guinney J. Zhao L. Zhang L. Hughes G. Sadda S. (2013). Genome-wide methylation profiles reveal quantitative views of human aging rates. *Mol. Cell* 49 359–367. 10.1016/j.molcel.2012.10.016 23177740 PMC3780611

[B88] Hayes R. B. (1997). The carcinogenicity of metals in humans. *Cancer Caus. Cont.* 8 371–385. 10.1023/a:1018457305212 9498900

[B89] He X. Qi Z. Hou H. Qian L. Gao J. Zhang X. X. (2020). Structural and functional alterations of gut microbiome in mice induced by chronic cadmium exposure. *Chemosphere* 246:125747. 10.1016/j.chemosphere.2019.125747 31891852

[B90] Hoelzer K. Wong N. Thomas J. Talkington K. Jungman E. Coukell A. (2017). Antimicrobial drug use in food-producing animals and associated human health risks: what, and how strong, is the evidence? *BMC Vet. Res.* 13:211. 10.1186/s12917-017-1131-3 28676125 PMC5496648

[B91] Hood S. Amir S. (2017a). Neurodegeneration and the Circadian Clock. *Front. Aging Neurosci.* 9:170. 10.3389/fnagi.2017.00170 PMC5447688 28611660

[B92] Hood S. Amir S. (2017b). The aging clock: circadian rhythms and later life. *J. Clin. Invest.* 127 437–446. 10.1172/JCI90328 28145903 PMC5272178

[B93] Hooper L. G. Kaufman J. D. (2018). Ambient air pollution and clinical implications for susceptible populations. *Ann. Am. Thorac. Soc.* 15 (Suppl. 2), S64–S68. 10.1513/AnnalsATS.201707-574MG 29676646 PMC5955035

[B94] Horvath S. D. N. A. (2013). methylation age of human tissues and cell types. *Genome Biol.* 14:R115. 10.1186/gb-2013-14-10-r115 PMC4015143 24138928

[B95] Huang H. Barzyk T. M. (2017). Connecting the dots: linking environmental justice indicators to daily dose model estimates. *Int. J. Environ. Res. Public Health* 2017:1. 10.3390/ijerph14010024 28036053 PMC5295275

[B96] Hunsberger H. C. Greenwood B. P. Tolstikov V. Narain N. R. Kiebish M. A. Denny C. A. (2020). Divergence in the metabolome between natural aging and Alzheimer’s disease. *Sci. Rep.* 10:12171. 10.1038/s41598-020-68739-z PMC7376199 32699218

[B97] Jackson C. L. Dagher R. K. Byun J. S. Farhat T. Gardner K. L. (2021). *Getting Under the Skin. The Science of Health Disparities Research.* Hoboken: Wiley, 13–38.

[B98] James K. A. Meliker J. R. (2013). Environmental cadmium exposure and osteoporosis: a review. *Int. J. Public Health* 58 737–745. 10.1007/s00038-013-0488-8 23877535

[B99] James K. A. Byers T. Hokanson J. E. Meliker J. R. Zerbe G. O. Marshall J. A. (2015). Association between lifetime exposure to inorganic arsenic in drinking water and coronary heart disease in Colorado residents. *Environ. Health Perspect* 123 128–134. 10.1289/ehp.1307839 25350952 PMC4314243

[B100] James K. A. Marshall J. A. Hokanson J. E. Meliker J. R. Zerbe G. O. Byers T. E. A. (2013). case-cohort study examining lifetime exposure to inorganic arsenic in drinking water and diabetes mellitus. *Environ. Res.* 123 33–38. 10.1016/j.envres.2013.02.005 23507312

[B101] Jan A. T. Azam M. Siddiqui K. Ali A. Choi I. Haq Q. M. R. (2015). Heavy Metals and human health: mechanistic insight into toxicity and counter defense system of antioxidants. *Int. J. Mol. Sci.* 16 29592–29630. 10.3390/ijms161226183 26690422 PMC4691126

[B102] Jardim M. J. (2011). microRNAs: implications for air pollution research. *Mutat. Res.* 717 38–45. 10.1016/j.mrfmmm.2011.03.014 21515291

[B103] Johnson M. S. Bailey T. L. Schmid K. K. Lydiatt W. M. Johanning J. M. A. (2014). frailty index identifies patients at high risk of mortality after tracheostomy. *Otolaryngol. Head Neck Surg.* 150 568–573. 10.1177/0194599813519749 24436464

[B104] Johnson P. A. Giles J. R. (2013). The hen as a model of ovarian cancer. *Nat. Rev. Cancer* 13 432–436. 10.1038/nrc3535 23676850

[B105] Jones K. E. Patel N. G. Levy M. A. Storeygard A. Balk D. Gittleman J. L. (2008). Global trends in emerging infectious diseases. *Nature* 451 990–993. 10.1038/nature06536 18288193 PMC5960580

[B106] Juarez P. D. Hood D. B. Song M. A. Ramesh A. (2020a). Use of an exposome approach to understand the effects of exposures from the natural, built, and social environments on cardio-vascular disease onset, progression, and outcomes. *Front. Public Health* 8:379. 10.3389/fpubh.2020.00379 32903514 PMC7437454

[B107] Juarez P. D. Matthews-Juarez P. Hood D. B. Im W. Levine R. S. Kilbourne B. J. (2014). The public health exposome: a population-based, exposure science approach to health disparities research. *Int. J. Environ. Res. Public Health* 11 12866–12895. 10.3390/ijerph111212866 25514145 PMC4276651

[B108] Juarez P. D. Tabatabai M. Valdez R. B. Hood D. B. Im W. Mouton C. (2020b). The effects of social, personal, and behavioral risk factors and PM2.5 on cardio-metabolic disparities in a cohort of community health center patients. *Int. J. Environ. Res. Public Health* 17:10. 10.3390/ijerph17103561 32438697 PMC7277630

[B109] Judge M. Griffith J. Arnold J. (2017). “Aging and the Biological Clock,” in *Circadian Rhythms and Their Impact on Aging*, eds Jazwinski S. M. Belancio V. P. Hill S. M. (Cham: Springer International Publishing), 211–234.

[B110] Khomenko S. Cirach M. Pereira-Barboza E. Mueller N. Barrera-Gómez J. Rojas-Rueda D. (2021). Premature mortality due to air pollution in European cities: a health impact assessment. *Lancet Planet Health* 5 e121–e134. 10.1016/S2542-5196(20)30272-2 33482109

[B111] Kilian J. Kitazawa M. (2018). The emerging risk of exposure to air pollution on cognitive decline and Alzheimer’s disease - Evidence from epidemiological and animal studies. *Biomed. J.* 41 141–162. 10.1016/j.bj.2018.06.001 30080655 PMC6138768

[B112] Kim S. Jazwinski S. M. (2018). The gut microbiota and healthy aging: a mini-review. *Gerontology* 64 513–520. 10.1159/000490615 30025401 PMC6191326

[B113] Kinirons M. T. O’Mahony M. S. (2004). Drug metabolism and ageing. *Br. J. Clin. Pharmacol.* 57 540–544. 15089805 10.1111/j.1365-2125.2004.02096.x PMC1884487

[B114] Kirkwood T. B. (2005). Understanding the odd science of aging. *Cell* 120 437–447. 10.1016/j.cell.2005.01.027 15734677

[B115] Knight A. K. Craig J. M. Theda C. Baekvad-Hansen M. Bybjerg-Grauholm J. Hansen C. S. (2016). An epigenetic clock for gestational age at birth based on blood methylation data. *Genome Biol.* 17:206. 10.1186/s13059-016-1068-z 27717399 PMC5054584

[B116] Kohanski R. A. Deeks S. G. Gravekamp C. Halter J. B. High K. Hurria A. (2016). Reverse geroscience: how does exposure to early diseases accelerate the age-related decline in health? *Ann. NY Acad. Sci.* 1386 30–44. 10.1111/nyas.13297 27907230

[B117] Krieger N. (2005). Embodiment: a conceptual glossary for epidemiology. *J. Epidemiol. Comm. Health* 59 350–355. 10.1136/jech.2004.024562 15831681 PMC1733093

[B118] Kulick E. R. Wellenius G. A. Boehme A. K. Joyce N. R. Schupf N. Kaufman J. D. (2020). Long-term exposure to air pollution and trajectories of cognitive decline among older adults. *Neurology* 94 e1782–e1792. 10.1212/WNL.0000000000009314 32269113 PMC7274848

[B119] Ladd-Acosta C. Feinberg J. I. Brown S. C. Lurmann F. W. Croen L. A. Hertz-Picciotto I. (2019). Epigenetic marks of prenatal air pollution exposure found in multiple tissues relevant for child health. *Environ. Int.* 126 363–376. 10.1016/j.envint.2019.02.028 30826615 PMC6446941

[B120] Lamont E. W. James F. O. Boivin D. B. Cermakian N. (2007). From circadian clock gene expression to pathologies. *Sleep Med.* 8 547–556. 10.1016/j.sleep.2006.11.002 17395534

[B121] Lee A. G. Cowell W. Kannan S. Ganguri H. B. Nentin F. Wilson A. (2020). Prenatal particulate air pollution and newborn telomere length: Effect modification by maternal antioxidant intakes and infant sex. *Environ. Res.* 187:109707. 10.1016/j.envres.2020.109707 32474316 PMC7844769

[B122] Lentini P. Zanoli L. Granata A. Signorelli S. S. Castellino P. Dell’Aquila R. (2017). Kidney and heavy metals - The role of environmental exposure (Review). *Mol. Med. Rep.* 15 3413–3419. 10.3892/mmr.2017.6389 28339049

[B123] Levine M. E. Lu A. T. Quach A. Chen B. H. Assimes T. L. Bandinelli S. (2018). An epigenetic biomarker of aging for lifespan and healthspan. *Aging* 10 573–591. 10.18632/aging.101414 29676998 PMC5940111

[B124] Lin Y. Damjanovic A. Metter E. J. Nguyen H. Truong T. Najarro K. (2015). Age-associated telomere attrition of lymphocytes in vivo is co-ordinated with changes in telomerase activity, composition of lymphocyte subsets and health conditions. *Clin. Sci.* 128 367–377. 10.1042/CS20140481 25317735 PMC5421624

[B125] Lind P. M. Salihovic S. Lind L. (2018). High plasma organochlorine pesticide levels are related to increased biological age as calculated by DNA methylation analysis. *Environ. Int.* 113 109–113. 10.1016/j.envint.2018.01.019 29421399

[B126] Liu T. Zhang L. Joo D. Sun S.-C. N. F. (2017). -κB signaling in inflammation. *Signal Transduct. Target Ther.* 2:17023. 10.1038/sigtrans.2017.23 PMC5661633 29158945

[B127] Lopez-Otin C. Blasco M. A. Partridge L. Serrano M. Kroemer G. (2013). The hallmarks of aging. *Cell* 153 1194–1217. 23746838 10.1016/j.cell.2013.05.039 PMC3836174

[B128] Lozupone C. A. Stombaugh J. I. Gordon J. I. Jansson J. K. Knight R. (2012). Diversity, stability and resilience of the human gut microbiota. *Nature* 489 220–230. 10.1038/nature11550 22972295 PMC3577372

[B129] Lu A. T. Quach A. Wilson J. G. Reiner A. P. Aviv A. Raj K. (2019). DNA methylation GrimAge strongly predicts lifespan and healthspan. *Aging* 11 303–327. 10.18632/aging.101684 30669119 PMC6366976

[B130] Lucchini R. G. Guazzetti S. Renzetti S. Conversano M. Cagna G. Fedrighi C. (2019). Neurocognitive impact of metal exposure and social stressors among schoolchildren in Taranto. *Italy. Environ. Health* 18:67. 10.1186/s12940-019-0505-3 31324194 PMC6642538

[B131] Luco R. F. Allo M. Schor I. E. Kornblihtt A. R. Misteli T. (2011). Epigenetics in alternative pre-mRNA splicing. *Cell* 144 16–26. 10.1016/j.cell.2010.11.056 21215366 PMC3038581

[B132] Ma S. Gladyshev V. N. (2017). Molecular signatures of longevity: Insights from cross-species comparative studies. *Semin. Cell Dev. Biol.* 70 190–203. 10.1016/j.semcdb.2017.08.007 28800931 PMC5807068

[B133] Mackenzie J. S. Jeggo M. (2019). The one health approach-why is it so important? *Trop. Med. Infect. Dis.* 4:88. 10.3390/tropicalmed4020088 31159338 PMC6630404

[B134] Mackenzie J. S. Smith D. W. (2020). 19: a novel zoonotic disease caused by a coronavirus from China: what we know and what we don’t. *Microbiol. Aust.* 41 45–50. 10.1071/MA20013 32226946 PMC7086482

[B135] Maierhofer A. Flunkert J. Oshima J. Martin G. M. Haaf T. Horvath S. (2017). Accelerated epigenetic aging in Werner syndrome. *Aging* 9 1143–1152. 10.18632/aging.101217 28377537 PMC5425119

[B136] Mainous A. G. III Wright R. U. Hulihan M. M. Twal W. O. McLaren C. E. Diaz V. A. (2014). Elevated transferrin saturation, health-related quality of life and telomere length. *Biometals* 27 135–141. 10.1007/s10534-013-9693-4 24337410 PMC4034347

[B137] Malecki K. Schultz A. Bergmans R. (2018). Neighborhood Perceptions and cumulative impacts of low level chronic exposure to fine particular matter (PM2.5) on Cardiopulmonary Health. *Int. J. Environ. Res. Public Health* 15:84. 10.3390/ijerph15010084 29316641 PMC5800183

[B138] Marini S. Davis K. A. Soare T. W. Zhu Y. Suderman M. J. Simpkin A. J. (2020). Adversity exposure during sensitive periods predicts accelerated epigenetic aging in children. *Psychoneuroendocrinology* 113:104484. 10.1016/j.psyneuen.2019.104484 31918390 PMC7832214

[B139] Martin C. L. Ward-Caviness C. K. Dhingra R. Zikry T. M. Galea S. Wildman D. E. (2021). Neighborhood environment, social cohesion, and epigenetic aging. *Aging* 13 7883–7899. 10.18632/aging.202814 33714950 PMC8034890

[B140] Martos S. N. Tang W. Y. Wang Z. (2015). Elusive inheritance: Transgenerational effects and epigenetic inheritance in human environmental disease. *Prog. Biophys. Mol. Biol.* 118 44–54. 10.1016/j.pbiomolbio.2015.02.011 25792089 PMC4784256

[B141] Mascarenhas M. Grattet R. Mege K. (2021). Toxic waste and race in twenty-first century america: neighborhood poverty and racial composition in the siting of hazardous waste facilities. *Environ. Soc.* 12 108–126.

[B142] McDermott M. M. Peterson C. A. Sufit R. Ferrucci L. Guralnik J. M. Kibbe M. R. (2018). Peripheral artery disease, calf skeletal muscle mitochondrial DNA copy number, and functional performance. *Vasc. Med.* 23 340–348. 10.1177/1358863X18765667 29734865 PMC6100735

[B143] McEwen B. S. Tucker P. (2011). Critical biological pathways for chronic psychosocial stress and research opportunities to advance the consideration of stress in chemical risk assessment. *Am. J. Public Health* 101 S131–S139. 10.2105/AJPH.2011.300270 22021312 PMC3222511

[B144] Michael Y. L. Yen I. H. (2014). Aging and place–neighborhoods and health in a world growing older. *J. Aging Health* 26 1251–1260. 10.1177/0898264314562148 25502240 PMC10339444

[B145] Milnerowicz H. Ściskalska M. Dul M. (2015). Pro-inflammatory effects of metals in persons and animals exposed to tobacco smoke. *J. Trace Elem. Med. Biol.* 29 1–10. 10.1016/j.jtemb.2014.04.008 24916792

[B146] Mirowsky J. E. Carraway M. S. Dhingra R. Tong H. Neas L. Diaz-Sanchez D. (2017). Ozone exposure is associated with acute changes in inflammation, fibrinolysis, and endothelial cell function in coronary artery disease patients. *Environ. Health* 16:126. 10.1186/s12940-017-0335-0 29157250 PMC5697214

[B147] Mitteldorf J. (2019). What is antagonistic pleiotropy? *Biochemistry* 84 1458–1468. 31870250 10.1134/S0006297919120058

[B148] Mohai P. Saha R. (2015). Which came first, people or pollution? A review of theory and evidence from longitudinal environmental justice studies. *Environ. Res. Lett.* 10:125011. 10.1088/1748-9326/10/12/125011

[B149] Mohai P. Pellow D. Roberts J. T. (2009). Environmental Justice. *Ann. Rev. Environ. Res.* 34 405–430.

[B150] Moon K. Guallar E. Navas-Acien A. (2012). Arsenic exposure and cardiovascular disease: an updated systematic review. *Curr. Atheroscler. Rep.* 14 542–555. 10.1007/s11883-012-0280-x 22968315 PMC3483370

[B151] Moore A. Z. Ding J. Tuke M. A. Wood A. R. Bandinelli S. Frayling T. M. (2018). Influence of cell distribution and diabetes status on the association between mitochondrial DNA copy number and aging phenotypes in the InCHIANTI study. *Aging Cell* 17:e12683. 10.1111/acel.12683 29047204 PMC5770782

[B152] Morello-Frosch R. Jesdale B. M. (2006). Separate and unequal: residential segregation and estimated cancer risks associated with ambient air toxics in U.S. metropolitan areas. *Environ. Health Perspect.* 114 386–393. 10.1289/ehp.8500 16507462 PMC1392233

[B153] Morello-Frosch R. Zuk M. Jerrett M. Shamasunder B. Kyle A. D. (2011). Understanding the cumulative impacts of inequalities in environmental health: implications for policy. *Health Aff.* 30 879–887. 10.1377/hlthaff.2011.0153 21555471

[B154] Munoz-Espin D. Serrano M. (2014). Cellular senescence: from physiology to pathology. *Nat. Rev. Mol. Cell Biol.* 15 482–496. 10.1038/nrm3823 24954210

[B155] Nagpal R. Mainali R. Ahmadi S. Wang S. Singh R. Kavanagh K. (2018). Gut microbiome and aging: Physiological and mechanistic insights. *Nut. Healthy Aging* 4 267–285. 10.3233/NHA-170030 29951588 PMC6004897

[B156] National Academy of Sciences (2014). “The National Academies Collection: Reports funded by National Institutes of Health,” in *Sociality, Hierarchy, Health: Comparative Biodemography: A Collection of Papers*, eds Weinstein M. Lane M. A. (Washington (DC): National Academies Press). 25254285

[B157] National Research Council (2013). *Critical Aspects of EPA’s IRIS Assessment of Inorganic Arsenic: Interim Report.* Washington, DC: National Research Council.

[B158] National Research Council (2014). *Sociality, Hierarchy, Health: Comparative Biodemography: A Collection of Papers.* Washington, DC: The National Academies Press. 25254285

[B159] Nawrot T. S. Saenen N. D. Schenk J. Janssen B. G. Motta V. Tarantini L. (2018). Placental circadian pathway methylation and in utero exposure to fine particle air pollution. *Environ. Int.* 114 231–241. 10.1016/j.envint.2018.02.034 29524919

[B160] Nawrot T. Plusquin M. Hogervorst J. Roels H. A. Celis H. Thijs L. (2006). Environmental exposure to cadmium and risk of cancer: a prospective population-based study. *Lancet Oncol.* 7 119–126. 10.1016/S1470-2045(06)70545-9 16455475

[B161] Nicoll R. (2018). Environmental contaminants and congenital heart defects: a re-evaluation of the evidence. *Int. J. Environ. Res. Public Health* 15:2096. 10.3390/ijerph15102096 30257432 PMC6210579

[B162] Niedernhofer L. J. Kirkland J. L. Ladiges W. (2017). Molecular pathology endpoints useful for aging studies. *Ageing Res. Rev.* 35 241–249. 10.1016/j.arr.2016.09.012 27721062 PMC5357461

[B163] Niedzwiecki M. M. Walker D. I. Vermeulen R. Chadeau-Hyam M. Jones D. P. Miller G. W. (2019). The exposome: molecules to populations. *Annu. Rev. Pharmacol. Toxicol.* 59 107–127. 10.1146/annurev-pharmtox-010818-021315 30095351

[B164] Nwanaji-Enwerem J. C. Colicino E. Trevisi L. Kloog I. Just A. C. Shen J. (2016). Long-term ambient particle exposures and blood DNA methylation age: findings from the VA normative aging study. *Environ. Epigenet.* 2:dvw006. 10.1093/eep/dvw006 27453791 PMC4957520

[B165] Nwanaji-Enwerem J. C. Jackson C. L. Ottinger M. A. Cardenas A. James K. A. Malecki K. M. C. (2021a). Adopting a compound exposome approach in environmental aging biomarker research: a call to action for advancing racial health equity. *Environ. Health Persp.* 129:045001. 10.1289/EHP8392 33822649 PMC8043128

[B166] Nwanaji-Enwerem J. C. Jenkins T. G. Colicino E. Cardenas A. Baccarelli A. A. Boyer E. W. (2020). Serum dioxin levels and sperm DNA methylation age: Findings in Vietnam war veterans exposed to Agent Orange. *Reprod. Toxicol.* 96 27–35. 10.1016/j.reprotox.2020.06.004 32522586

[B167] Nwanaji-Enwerem J. C. Laan L. V. Avakame E. F. Scott K. A. Burris H. H. Cardenas A. (2021b). Associations of DNA methylation mortality risk markers with congenital microcephaly from zika virus: a study of brazilian children less than 4 years of age. *J. Trop. Pediatr.* 67:fmab020. 10.1093/tropej/fmab020 33822234 PMC8022928

[B168] Ogburn C. E. Carlberg K. Ottinger M. A. Holmes D. J. Martin G. M. Austad S. N. (2001). Exceptional cellular resistance to oxidative damage in long-lived birds requires active gene expression. *J. Gerontol. A Biol. Sci. Med. Sci.* 56 B468–B474. 10.1093/gerona/56.11.b468 11682567

[B169] Ogojiaku C. N. Allen J. C. Anson-Dwamena R. Barnett K. S. Adetona O. Im W. (2020). The health opportunity index: understanding the input to disparate health outcomes in vulnerable and high-risk census tracts. *Int. J. Environ. Res. Public Health* 17:16. 10.3390/ijerph17165767 32785046 PMC7459470

[B170] Olvera Alvarez H. A. Appleton A. A. Fuller C. H. Belcourt A. Kubzansky L. D. (2018). An integrated socio-environmental model of health and well-being: a conceptual framework exploring the joint contribution of environmental and social exposures to health and disease over the life span. *Curr. Environ. Health Rep.* 5 233–243. 10.1007/s40572-018-0191-2 29574677

[B171] Ottinger M. A. (2007). Neuroendocrine aging in birds: comparing lifespan differences and conserved mechanisms. *Ageing Res. Rev.* 6 46–53. 10.1016/j.arr.2007.02.006 17452025

[B172] Ottinger M. A. Abdelnabi M. Li Q. Chen K. Thompson N. Harada N. (2004). The Japanese quail: a model for studying reproductive aging of hypothalamic systems. *Exp. Gerontol.* 39 1679–1693. 10.1016/j.exger.2004.06.021 15582284

[B173] Ottinger M. A. Lavoie E. (2007). Neuroendocrine and immune characteristics of aging in avian species. *Cytogenet. Genome Res.* 117 352–357. 10.1159/000103198 17675878

[B174] Pal S. Tyler J. K. (2016). Epigenetics and aging. *Sci. Adv.* 2:e1600584. 10.1126/sciadv.1600584 PMC4966880 27482540

[B175] Park M. Verhoeven J. E. Cuijpers P. Reynolds Iii C. F. Penninx B. W. J. H. (2015). Where you live may make you old: the association between perceived poor neighborhood quality and leukocyte telomere length. *PLoS One* 10:e0128460. 10.1371/journal.pone.0128460 26083263 PMC4471265

[B176] Pembrey M. Saffery R. Bygren L. O. Network in Epigenetic E. (2014). Human transgenerational responses to early-life experience: potential impact on development, health and biomedical research. *J. Med. Genet.* 51 563–572. 10.1136/jmedgenet-2014-102577 25062846 PMC4157403

[B177] Peng C. Cardenas A. Rifas-Shiman S. L. Hivert M. F. Gold D. R. Platts-Mills T. A. (2019). Epigenetic age acceleration is associated with allergy and asthma in children in Project Viva. *J. Allergy Clin. Immunol.* 143:2263. 10.1016/j.jaci.2019.01.034 30738172 PMC6556426

[B178] Peters A. Nawrot T. S. Baccarelli A. A. (2021). Hallmarks of environmental insults. *Cell* 184 1455–1468. 10.1016/j.cell.2021.01.043 33657411 PMC9396710

[B179] Petroni M. Hill D. Younes L. Barkman L. Howard S. Howell I. B. (2020). Hazardous air pollutant exposure as a contributing factor to COVID-19 mortality in the United States. *Environ. Res. Lett.* 15:940.

[B180] Pezzoli G. Cereda E. (2013). Exposure to pesticides or solvents and risk of Parkinson disease. *Neurology* 80 2035–2041. 10.1212/WNL.0b013e318294b3c8 23713084

[B181] Pierce B. L. Tong L. Dean S. Argos M. Jasmine F. Rakibuz-Zaman M. (2019). A missense variant in FTCD is associated with arsenic metabolism and toxicity phenotypes in Bangladesh. *PLoS Genet.* 15:e1007984. 10.1371/journal.pgen.1007984 PMC6443193 30893314

[B182] Power M. C. Korrick S. Tchetgen Tchetgen E. J. Nie L. H. Grodstein F. Hu H. (2014). Lead exposure and rate of change in cognitive function in older women. *Environ. Res.* 129 69–75. 10.1016/j.envres.2013.12.010 24529005 PMC3951744

[B183] Puterman E. Lin J. Krauss J. Blackburn E. H. Epel E. S. (2015). Determinants of telomere attrition over 1 year in healthy older women: stress and health behaviors matter. *Mol. Psychiatry* 20 529–535. 10.1038/mp.2014.70 25070535 PMC4310821

[B184] Queiroz J. D. N. Macedo R. C. O. Tinsley G. M. Reischak-Oliveira A. (2020). Time-restricted eating and circadian rhythms: the biological clock is ticking. *Crit. Rev. Food Sci. Nutr.* 2020 1–13. 10.1080/10408398.2020.1789550 32662279

[B185] Ragonnaud E. Biragyn A. (2021). Gut microbiota as the key controllers of “healthy” aging of elderly people. *Immun. Ageing* 18:2. 10.1186/s12979-020-00213-w 33397404 PMC7784378

[B186] Rodier F. Campisi J. (2011). Four faces of cellular senescence. *J. Cell Biol.* 192 547–556. 10.1083/jcb.201009094 21321098 PMC3044123

[B187] Roenneberg T. Kumar C. J. Merrow M. (2007). The human circadian clock entrains to sun time. *Curr. Biol.* 17 R44–R45. 10.1016/j.cub.2006.12.011 17240323

[B188] Roy A. Georgopoulos P. G. Ouyang M. Freeman N. Lioy P. J. (2003). Environmental, dietary, demographic, and activity variables associated with biomarkers of exposure for benzene and lead. *J. Expo. Anal. Environ. Epidemiol.* 13 417–426. 10.1038/sj.jea.7500296 14603342

[B189] Saha D. T. Davidson B. J. Wang A. Pollock A. J. Orden R. A. Goldman R. (2008). Quantification of DNA repair capacity in whole blood of patients with head and neck cancer and healthy donors by comet assay. *Mutat. Res.* 650 55–62. 10.1016/j.mrgentox.2007.10.004 18032094 PMC4289908

[B190] Saldmann F. Viltard M. Leroy C. Friedlander G. (2019). The naked mole rat: a unique example of positive oxidative stress. *Oxid. Med. Cell Longev.* 2019:4502819. 10.1155/2019/4502819 30881592 PMC6383544

[B191] Scheiermann C. Kunisaki Y. Frenette P. S. (2013). Circadian control of the immune system. *Nat. Rev. Immunol.* 13 190–198. 10.1038/nri3386 23391992 PMC4090048

[B192] Schikowski T. Altuğ H. (2020). The role of air pollution in cognitive impairment and decline. *Neurochem. Int.* 136 104708. 10.1016/j.neuint.2020.104708 32092328

[B193] Schmidt C. Peigneux P. Cajochen C. (2012). Age-Related changes in sleep and circadian rhythms: impact on cognitive performance and underlying neuroanatomical networks. *Front. Neurol.* 3:118. 10.3389/fneur.2012.00118 22855682 PMC3405459

[B194] Searle S. D. Mitnitski A. Gahbauer E. A. Gill T. M. Rockwood K. A. (2008). standard procedure for creating a frailty index. *BMC Geriat.* 8:24. 10.1186/1471-2318-8-24 PMC2573877 18826625

[B195] Sehl M. E. Henry J. E. Storniolo A. M. Ganz P. A. Horvath S. D. N. A. (2017). methylation age is elevated in breast tissue of healthy women. *Breast Cancer Res. Treat.* 164 209–219. 10.1007/s10549-017-4218-4 28364215 PMC5487725

[B196] Sharma M. Li Y. Stoll M. L. Tollefsbol T. O. (2020). The epigenetic connection between the gut microbiome in obesity and diabetes. *Front. Genet.* 10:1329. 10.3389/fgene.2019.01329 32010189 PMC6974692

[B197] Shiels P. G. Buchanan S. Selman C. Stenvinkel P. (2019). Allostatic load and ageing: linking the microbiome and nutrition with age-related health. *Biochem. Soc. Trans.* 47 1165–1172. 10.1042/BST20190110 31416886

[B198] Shiels P. G. Painer J. Natterson-Horowitz B. Johnson R. J. Miranda J. J. Stenvinkel P. (2021). Manipulating the exposome to enable better ageing. *Biochem. J.* 478 2889–2898. 10.1042/BCJ20200958 34319404 PMC8331090

[B199] Sierra F. (2016a). Moving geroscience into uncharted waters. *J. Gerontol. A Biol. Sci. Med. Sci.* 71 1385–1387. 10.1093/gerona/glw087 27535965

[B200] Sierra F. (2016b). The emergence of geroscience as an interdisciplinary approach to the enhancement of health span and life span. *Cold Spring Harb. Persp. Med.* 6:a025163. 10.1101/cshperspect.a025163 26931460 PMC4817738

[B201] Sitzmann B. D. Lemos D. R. Ottinger M. A. Urbanski H. F. (2010). Effects of age on clock gene expression in the rhesus macaque pituitary gland. *Neurobiol. Aging* 31 696–705. 10.1016/j.neurobiolaging.2008.05.024 18614257 PMC2823945

[B202] Smith A. Kaufman F. Sandy M. S. Cardenas A. (2020). Cannabis exposure during critical windows of development: epigenetic and molecular pathways implicated in neuropsychiatric disease. *Curr. Environ. Health Rep.* 7 325–342. 10.1007/s40572-020-00275-4 32441004 PMC7458902

[B203] Song S. Lam E. W. Tchkonia T. Kirkland J. L. Sun Y. (2020). Senescent cells: emerging targets for human aging and age-related diseases. *Trends Biochem. Sci.* 45 578–592. 10.1016/j.tibs.2020.03.008 32531228 PMC7649645

[B204] Sternberg S. A. Wershof Schwartz A. Karunananthan S. Bergman H. Mark Clarfield A. (2011). The identification of frailty: a systematic literature review. *J. Am. Geriatr. Soc.* 59 2129–2138. 10.1111/j.1532-5415.2011.03597.x 22091630

[B205] Swanson J. M. Entringer S. Buss C. Wadhwa P. D. (2009). Developmental origins of health and disease: environmental exposures. *Semin. Reprod. Med.* 27 391–402. 10.1055/s-0029-1237427 19711249 PMC2862627

[B206] Swanson K. V. Deng M. Ting J. P. (2019). The NLRP3 inflammasome: molecular activation and regulation to therapeutics. *Nat. Rev. Immunol.* 19 477–489. 10.1038/s41577-019-0165-0 31036962 PMC7807242

[B207] Tellez-Plaza M. Guallar E. Howard B. V. Umans J. G. Francesconi K. A. Goessler W. (2013). Cadmium exposure and incident cardiovascular disease. *Epidemiology* 24 421–429. 10.1097/EDE.0b013e31828b0631 23514838 PMC4142588

[B208] Tonne C. Elbaz A. Beevers S. Singh-Manoux A. (2014). Traffic-related Air pollution in relation to cognitive function in older adults. *Epidemiology* 25 674–681. 10.1097/EDE.0000000000000144 25036434 PMC4162337

[B209] Tsamou M. Vrijens K. Madhloum N. Lefebvre W. Vanpoucke C. Nawrot T. S. (2018). Air pollution-induced placental epigenetic alterations in early life: a candidate miRNA approach. *Epigenetics* 13 135–146. 10.1080/15592294.2016.1155012 27104955 PMC5873362

[B210] Tu P. Chi L. Bodnar W. Zhang Z. Gao B. Bian X. (2020). Gut microbiome toxicity: connecting the environment and gut microbiome-associated diseases. *Toxics* 8:19. 10.3390/toxics8010019 32178396 PMC7151736

[B211] Urbanski H. F. Sorwell K. G. (2012). Age-related changes in neuroendocrine rhythmic function in the rhesus macaque. *Age* 34 1111–1121. 10.1007/s11357-011-9352-z 22198672 PMC3448984

[B212] Vanleerberghe P. De Witte N. Claes C. Schalock R. L. Verte D. (2017). The quality of life of older people aging in place: a literature review. *Qual. Life Res.* 26 2899–2907. 10.1007/s11136-017-1651-0 28707047

[B213] Vaz Fragoso C. A. Manini T. M. Kairalla J. A. Buford T. W. Hsu F. C. Gill T. M. (2019). Mitochondrial DNA variants and pulmonary function in older persons. *Exp. Gerontol.* 115 96–103. 10.1016/j.exger.2018.11.023 30508565 PMC6356103

[B214] Vermeulen R. Schymanski E. L. Barabasi A. L. Miller G. W. (2020). The exposome and health: Where chemistry meets biology. *Science* 367 392–396. 10.1126/science.aay3164 31974245 PMC7227413

[B215] Vriens A. Nawrot T. S. Janssen B. G. Baeyens W. Bruckers L. Covaci A. (2019). Exposure to environmental pollutants and their association with biomarkers of aging: a multipollutant approach. *Environ. Sci. Technol.* 53 5966–5976. 10.1021/acs.est.8b07141 31041867

[B216] Wadhwa P. D. Buss C. Entringer S. Swanson J. M. (2009). Developmental origins of health and disease: brief history of the approach and current focus on epigenetic mechanisms. *Semin. Reprod. Med.* 27 358–368. 10.1055/s-0029-1237424 19711246 PMC2862635

[B217] Walker D. I. Valvi D. Rothman N. Lan Q. Miller G. W. Jones D. P. (2019). The metabolome: A key measure for exposome research in epidemiology. *Curr. Epidemiol. Rep.* 6 93–103. 10.1007/s40471-019-00187-4 31828002 PMC6905435

[B218] Walston J. McBurnie M. A. Newman A. Tracy R. P. Kop W. J. Hirsch C. H. (2002). Frailty and activation of the inflammation and coagulation systems with and without clinical comorbidities: results from the Cardiovascular Health Study. *Arch. Intern. Med.* 162 2333–2341. 10.1001/archinte.162.20.2333 12418947

[B219] Wan H. Wang Q. Chen X. Zeng Q. Shao Y. Fang H. (2020). WDR45 contributes to neurodegeneration through regulation of ER homeostasis and neuronal death. *Autophagy* 16 531–547. 10.1080/15548627.2019.1630224 31204559 PMC6999610

[B220] Wang M. Liang B. Zhang W. Chen K. Zhang Y. Zhou H. (2019). Dietary Lead exposure and associated health risks in guangzhou, China. *Int. J. Environ. Res. Public Health* 16:8. 10.3390/ijerph16081417 31010248 PMC6517897

[B221] Ward-Caviness C. K. Nwanaji-Enwerem J. C. Wolf K. Wahl S. Colicino E. Trevisi L. (2016). Long-term exposure to air pollution is associated with biological aging. *Oncotarget* 7 74510–74525. 10.18632/oncotarget.12903 27793020 PMC5342683

[B222] Ward-Caviness C. K. Pu S. Martin C. L. Galea S. Uddin M. Wildman D. E. (2020a). Epigenetic predictors of all-cause mortality are associated with objective measures of neighborhood disadvantage in an urban population. *Clin. Epigen.* 12:44. 10.1186/s13148-020-00830-8 32160902 PMC7065313

[B223] Ward-Caviness C. K. Russell A. G. Weaver A. M. Slawsky E. Dhingra R. Kwee L. C. (2020b). Accelerated epigenetic age as a biomarker of cardiovascular sensitivity to traffic-related air pollution. *Aging* 12 24141–24155. 10.18632/aging.202341 33289704 PMC7762491

[B224] Weaver A. M. McGuinn L. A. Neas L. Devlin R. B. Dhingra R. Ward-Caviness C. K. (2022). Associations between neighborhood socioeconomic cluster and hypertension, diabetes, myocardial infarction, and coronary artery disease within a cohort of cardiac catheterization patients. *Am. Heart J.* 243 201–209. 10.1016/j.ahj.2021.09.013 34610283 PMC8633144

[B225] Weaver A. Pfaff E. Neas L. Devlin R. Diaz-Sanchez D. WardCaviness C. (2019). Neighborhood socioeconomic status and mortality among heart failure patients. *Environ. Epidemiol.* 3:429.

[B226] Wehrens S. M. T. Christou S. Isherwood C. Middleton B. Gibbs M. A. Archer S. N. (2017). Meal timing regulates the human circadian system. *Curr. Biol.* 27 1768–1775. 10.1016/j.cub.2017.04.059 28578930 PMC5483233

[B227] White A. J. Kresovich J. K. Keller J. P. Xu Z. Kaufman J. D. Weinberg C. R. (2019). Air pollution, particulate matter composition and methylation-based biologic age. *Environ. Int.* 132:105071. 10.1016/j.envint.2019.105071 31387022 PMC6754788

[B228] Wild C. P. (2012). The exposome: from concept to utility. *Int. J. Epidemiol.* 41 24–32. 10.1093/ije/dyr236 22296988

[B229] Wild C. P. Scalbert A. Herceg Z. (2013). Measuring the exposome: a powerful basis for evaluating environmental exposures and cancer risk. *Environ. Mol. Mutagen.* 54 480–499. 10.1002/em.21777 23681765

[B230] Wilmanski T. Diener C. Rappaport N. Patwardhan S. Wiedrick J. Lapidus J. (2021). Gut microbiome pattern reflects healthy ageing and predicts survival in humans. *Nat. Metabol.* 3 274–286. 10.1038/s42255-021-00348-0 PMC8169080 33619379

[B231] Wilson I. D. Nicholson J. K. (2017). Gut microbiome interactions with drug metabolism, efficacy, and toxicity. *Transl. Res.* 179 204–222. 10.1016/j.trsl.2016.08.002 27591027 PMC5718288

[B232] Won J. Lee C. Forjuoh S. N. Ory M. G. (2016). Neighborhood safety factors associated with older adults’ health-related outcomes: A systematic literature review. *Soc. Sci. Med.* 165 177–186. 10.1016/j.socscimed.2016.07.024 27484353

[B233] Wright R. O. (2017). Environment, susceptibility windows, development, and child health. *Curr. Opin. Pediatr.* 29 211–217. 10.1097/MOP.0000000000000465 28107208 PMC5473288

[B234] Wu H. C. Tseng M. H. (2018). Evaluating disparities in elderly community care resources: using a geographic accessibility and inequality index. *Int. J. Environ. Res. Public Health* 15:7. 10.3390/ijerph15071353 29954156 PMC6068710

[B235] Wu X. Nethery R. C. Sabath B. M. Braun D. Dominici F. (2020). Exposure to air pollution and COVID-19 mortality in the United States: a nationwide cross-sectional study. *medRxiv* 10.1101/2020.04.05.20054502 33148655 PMC7673673

[B236] Xiao L. Zan G. Feng X. Bao Y. Huang S. Luo X. (2021). The associations of multiple metals mixture with accelerated DNA methylation aging. *Environ. Pollut.* 269:116230. 10.1016/j.envpol.2020.116230 33316491

[B237] Younan D. Wang X. Casanova R. Barnard R. Gaussoin S. A. Saldana S. (2021). PM associated with gray matter atrophy reflecting increased alzheimer risk in older women. *Neurology* 96 e1190–e1201. 10.1212/WNL.0000000000011149 33208540 PMC8055348

[B238] Zawia N. H. (2003). Transcriptional involvement in neurotoxicity. *Toxicol. Appl. Pharmacol.* 190 177–188. 10.1016/s0041-008x(03)00161-3 12878047

[B239] Zhang C. Kibriya M. G. Jasmine F. Roy S. Gao J. Sabarinathan M. (2018). A study of telomere length, arsenic exposure, and arsenic toxicity in a Bangladeshi cohort. *Environ. Res.* 164 346–355. 10.1016/j.envres.2018.03.005 29567420 PMC6647858

[B240] Zhang F. Kerbl-Knapp J. Akhmetshina A. Korbelius M. Kuentzel K. B. Vujiæ N. (2021). Tissue-specific landscape of metabolic dysregulation during ageing. *Biomolecules* 11:235. 10.3390/biom11020235 33562384 PMC7914945

[B241] Zhang H. Puleston D. J. Simon A. K. (2016). Autophagy and immune senescence. *Trends Mol. Med.* 22 671–686. 10.1016/j.molmed.2016.06.001 27395769

[B242] Zhang R. Wang Y. Ye K. Picard M. Gu Z. (2017). Independent impacts of aging on mitochondrial DNA quantity and quality in humans. *BMC Genomics* 18:890. 10.1186/s12864-017-4287-0 29157198 PMC5697406

[B243] Zhang X. Chen X. Zhang X. (2018). The impact of exposure to air pollution on cognitive performance. *Proc. Nat. Acad. Sci.* 115 9193–9197. 10.1073/pnas.1809474115 30150383 PMC6140474

[B244] Zhong J. Karlsson O. Wang G. Li J. Guo Y. Lin X. (2017). B vitamins attenuate the epigenetic effects of ambient fine particles in a pilot human intervention trial. *Proc. Nat. Acad. Sci.* 114 3503–3508. 28289216 10.1073/pnas.1618545114 PMC5380085

[B245] Zota A. R. Needham B. L. Blackburn E. H. Lin J. Park S. K. Rehkopf D. H. (2015). Associations of cadmium and lead exposure with leukocyte telomere length: findings from National Health and Nutrition Examination Survey, 1999-2002. *Am. J. Epidemiol.* 181 127–136. 10.1093/aje/kwu293 25504027 PMC4351349

